# I_NaP_ selective inhibition reverts precocious inter- and motorneurons hyperexcitability in the Sod1-G93R zebrafish ALS model

**DOI:** 10.1038/srep24515

**Published:** 2016-04-15

**Authors:** Lorena Benedetti, Anna Ghilardi, Elsa Rottoli, Marcella De Maglie, Laura Prosperi, Carla Perego, Mirko Baruscotti, Annalisa Bucchi, Luca Del Giacco, Maura Francolini

**Affiliations:** 1 Department of Medical Biotechnology and Translational Medicine, University of Milan, Neuroscience Institute, National Research Council (CNR), Via Vanvitelli 32, 20139 Milano, Italy; 2 Department of BioSciences, University of Milan, Via Celoria 26, 20133 Milano, Italy; 3 Department of Veterinary Science and Public Health, University of Milan, Via Celoria 10, 20133 Milano, Italy; 4 Department of Pharmacological and Biomolecular Sciences, University of Milan, Via Trentacoste 2, 20133 Milano, Italy

## Abstract

The pathogenic role of *SOD1* mutations in amyotrophic lateral sclerosis (ALS) was investigated using a zebrafish disease model stably expressing the ALS-linked G93R mutation. In addition to the main pathological features of ALS shown by adult fish, we found remarkably precocious alterations in the development of motor nerve circuitry and embryo behavior, and suggest that these alterations are prompted by interneuron and motor neuron hyperexcitability triggered by anomalies in the persistent pacemaker sodium current I_NaP_. The riluzole-induced modulation of I_NaP_ reduced spinal neuron excitability, reverted the behavioral phenotypes and improved the deficits in motor nerve circuitry development, thus shedding new light on the use of riluzole in the management of ALS. Our findings provide a valid phenotype-based tool for unbiased *in vivo* drug screening that can be used to develop new therapies.

Amyotrophic lateral sclerosis (ALS) is a highly debilitating disease caused by the progressive degeneration of upper and lower motor neurons. This gradual failure of the neuromuscular system leads to progressive paralysis and, eventually, death due to respiratory failure^1^. ALS may be sporadic (SALS, about 90% of cases) or familial (FALS, about 10%), and mutations in the *SOD1* gene account for 12–20% of the cases of FALS and 1–7% of the cases of SALS^2^,^3^,^4^. There is currently no cure, although the drug riluzole can prolong the life of a subset of patients by 2–4 months, albeit without reducing the symptoms^5^.

As it is not possible to take repeated real-time biopsy samples of nervous tissue from ALS patients safely, it is difficult to clarify how, why and when motor neurons are damaged in the various stages of the disease. Moreover, as almost all autoptic samples come from end-stage patients, they are not very useful for developing new therapies. Consequently, the use of animal models is the only means of studying cell and molecular processes, identifying key pathways for intervention, and assessing multiple candidate therapies over short periods of time. Rodent^6^, fruit fly^7^ and nematode worm models^8^ have been used to investigate the neurobiological basis of ALS, and have shown that it is a non-cell-autonomous multifactorial disease in which a whole range of cell types could contribute to the maintenance of motor neurons^9^.

The high degree of conservation of the genes involved in neurodegenerative diseases makes zebrafish a powerful means of studying nervous system disorders^10^,^11^, the molecular, electrophysiological, and behavioral aspects of which can be revealed by the fish’s stereotypical movement patterns and tactile responses^12^,^13^. Furthermore, the discovery that their spontaneous alternating side-to-side trunk contractions (coiling), which start 17 hours post-fertilization (hpf), are not myogenic and solely originate from the activity of three types of interneurons and the commissural primary ascending motor neurons is particularly relevant^12^,^14^,^15^. These neurons show rhythmic membrane potential oscillations (periodic depolarizations giving rise to spontaneous coiling), which are insensitive to the neurotransmitter receptor blockade^14^ being dependent on the persistent sodium current I_NaP_. The administration of 5 μM riluzole abolishes the periodic depolarizations, inhibiting I_NaP_ and, as the same amount also blocks coiling, it has been suggested that I_NaP_ drives coiling activity^16^.

Transgenic G93R (mSod1) and wild-type (wtSod1) zebrafish are respectively characterized by the ubiquitous over-expression of G93R mutated Sod1 and the wild-type Sod1^17^; the G93R substitution affects an evolutionarily conserved amino acid that is mutated in FALS^18^. After characterizing the disease phenotype in adult zebrafish over-expressing mSod1 or wtSod1 by studying all of the main pathological events occurring in ALS, we looked for precocious hallmarks of ALS in transgenic embryos/larvae by analyzing the development of spinal motor neurons and the maturation of neuromuscular junctions (NMJ). These analyses were paralleled by behavioral tests designed to associate changes in motor activity with specific anatomical defects in neuromuscular connections. Finally we revealed and pharmacologically modulated alterations in the electrical properties of mSod1 spinal neurons.

## Results

### Adult Sod1 G93R zebrafish show the typical neuromuscular features of ALS

As the clinical signatures of ALS (i.e. weakness and muscular atrophy) appear in adulthood, we searched for signs of motor impairment associated with disease progression by studying spontaneous locomotor activity in 12-month-old zebrafish. Analyses of their swimming movements showed that the mSod1 fish covered a significantly shorter distance and rested for significantly longer than the wtSod1 and control (Ctrl) fish, whereas there was no difference in the swimming speed of the three genotypes (Fig. S1).

**Figure 1:**
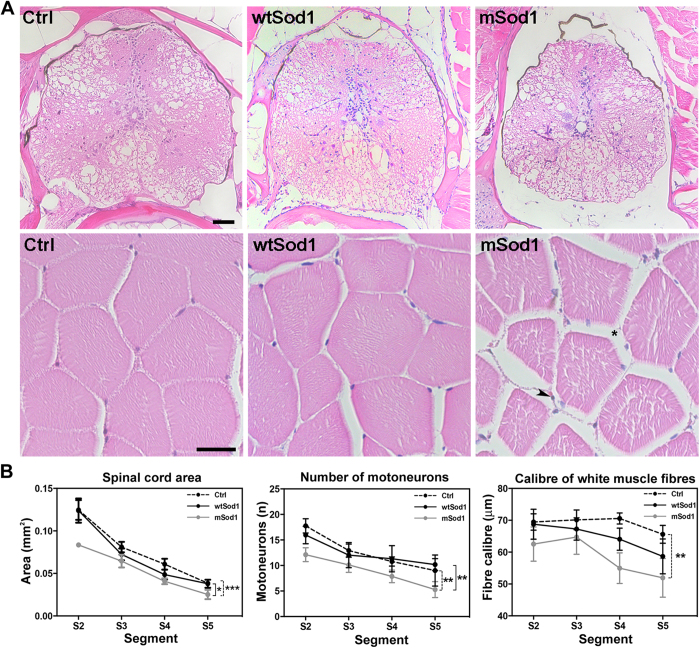
Spinal cord and lateral muscle histological analyses. Each adult fish was transversely cut into five segments (S1–S5) using the fins as anatomical references (see Supp. Figure S2). **(A)** Hematoxylin & eosin-stained histological sections of the S2 segment of the spinal cord (upper panels; scale bar: 50 μm) and white muscle fibres (lower panels; scale bar: 25 μm) in control (Ctrl), wtSod1 and mSod1 zebrafish. Muscular atrophy and edema (*) with infiltrating cells (arrowhead) are visible in mSod1 lateral muscle. **(B)** The plots show the significant reduction in spinal cord area and the number of motor neurons throughout the spinal cord, and a significant reduction in the calibre of white muscle fibres along the mSod1 fish trunk. Each point in the plots shows the mean value ± SEM of the indicated parameter in each segment of seven animals for each genotype. The measures were statistically analyzed using two-way ANOVA, and corrected by means of Sidak’s multiple comparison test (*P < 0.05; **P < 0.01; ***P < 0.001).

**Figure 2:**
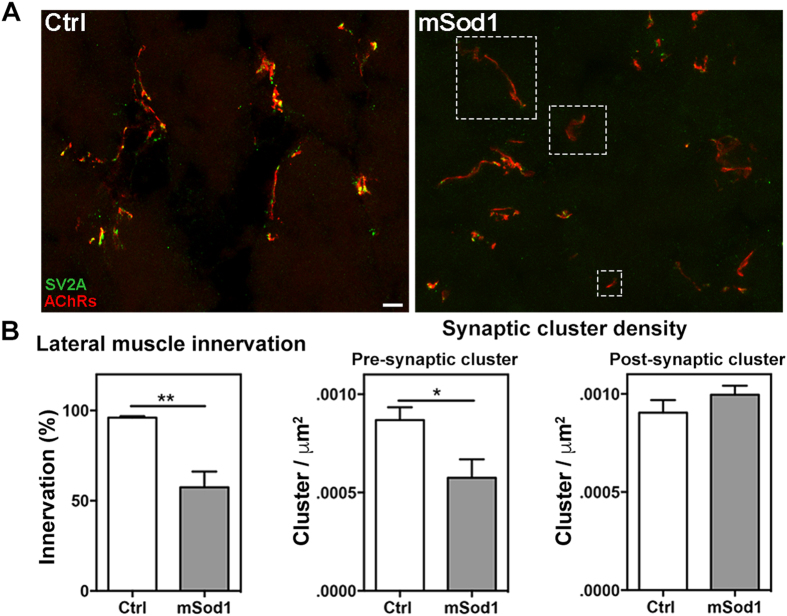
Twelve-month-old mSod1 zebrafish show compromised lateral white muscle innervation. **(A)** Maximum projections of confocal images of synaptic vesicle protein 2A (SV2A, green) and muscle acetylcholine receptors (AChRs, red) covering the entire thickness (20 μm) of a Ctrl and mSod1 zebrafish lateral muscle cryostat section. In the Ctrl zebrafish, each post-synaptic specialization enriched with acetylcholine receptors faces motor nerve terminals containing vesicle clusters, whereas many of the post-synaptic clusters in the mSod1 section lack an association with motor pre-synaptic terminals (white dashed boxes). Scale bar: 20 μm. **(B)** The percentage of innervation of post-synaptic specializations is significantly reduced in the mSod1 zebrafish (57.48 ± 8.69% *vs* 95.94 ± 0.84%), and three-dimensional co-localization analysis of z-stacks covering the entire thickness of the sections revealed a significant reduction in pre-synaptic cluster density (5.76 ± 0.09 *vs* 8.69 ± 0.06 × 10^−4^ clusters/μm^2^) but not in post-synaptic cluster density (9.96 ± 0.05 *vs* 9.04 ± 0.06 × 10^−4^ clusters/μm^2^). The columns in each graph indicate the mean value ± SEM of the indicated parameter in five Ctrl and six mSod1 zebrafish. The measures were statistically analyzed using an unpaired Student *t*-test (*P < 0.05; **P < 0.01).

As there were no differences in the external macroscopic features of the three groups (Fig. S2), we looked for possible motor neuron loss and spinal cord and muscle atrophy in the trunk of the transgenic fish (Fig. 1), and found a significant reduction in spinal cord area and the number of motor neurons in mSod1, and a substantial decrease in mean white fibre calibre, which was particularly severe in the most caudal portion of the body (Fig. 1). White muscle innervation was evaluated by fluorescence staining (Fig. 2A) as the percentage of post-synaptic clusters facing pre-synaptic vesicle clusters, after which the density of pre- and post-synaptic clusters was measured in lateral muscle. There was a significant reduction in the percentage of mSod1 white lateral muscle innervation, but only the pre-synaptic puncta showed a significant decrease in density (Fig. 2B); the fluorescence intensity and size of both the pre- and post-synaptic clusters were unaffected (Fig. S3A). Despite the extent of denervation, ultrastructural analyses of the remaining NMJs showed that the pre-synaptic boutons in the lateral muscle of mSod1 were identical to those observed in control, and there were no alterations in the fine structural organization of sarcomeres (Fig. S3B).

### Adult mSod1 zebrafish present reactive astrogliosis in the spinal cord and activated inflammatory cells infiltrate lateral muscles

As reactive astrogliosis and microgliosis are specific hallmarks of ALS in patients and rodent models, we analyzed the distribution of GFAP and Aif1 in adult fish (Fig. S4). The GFAP signal was significantly stronger in the spinal cord of wtSod1 and mSod1, and particularly pronounced in the last segment of mSod1, but no changes were detected in the Aif1 signal (Fig. S4A). Finally, the lateral muscles of the trunk showed a significant increase in Aif1 labelling, thus implying the presence of inflammatory cells in this district (Fig. S4B).

### Mutant Sod1 expression precociously affects locomotor network architecture

As mSod1 adults present the hallmarks of ALS, we used whole embryo fluorescence staining to compare the developing locomotor network of Ctrl, wtSod1 and mSod1 embryos and larvae (Fig. S5) in a search for the alterations associated with the earliest stages of the disease.

**Figure 3:**
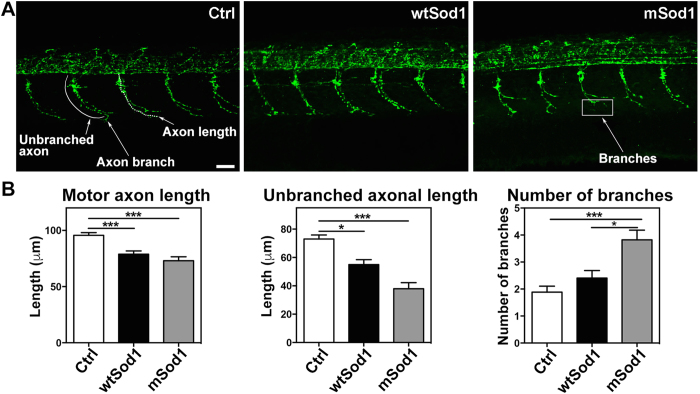
Sod1 overexpression causes motor nerve alterations at 24 hpf. **(A)** Confocal fluorescence maximum projection images showing SV2A signals (green) in the 12–16^th^ somite region of the entire trunk of control (Ctrl), wtSod1 and mSod1 zebrafish embryos at 24 hpf (the same analysis was made in the 17–21^st^ somite region with comparable results, data not shown). As synaptic vesicles travel the entire axonal length, it is possible to observe the length of embryonal motor axons and motor nerve branches. Scale bar: 25 μm. **(B)** Both wtSod1 (78.9 ± 2.8 μm) and mSod1 embryos (73.1 ± 3.5 μm) showed significantly shorter motor axons than the Ctrl (95.6 ± 2.4 μm) and a significant decrease in unbranched axonal length (55.0 ± 3.5 and 38.0 ± 4.3 μm *vs* 73.0 ± 2.8 μm), but only the mSod1 embryos showed a significant increase in the number of motor nerve branches: 3.8 ± 0.4 *vs* 1.9 ± 0.2 (Ctrl) and 2.4 ± 0.3 (wtSod1). The columns indicate the mean value ± SEM of the indicated parameter in at least five motor nerves of each of 25 Ctrl, 17 wtSod1, and 21 mSod1 embryos. The measures were statistically analyzed using one-way ANOVA or the Kruskal-Wallis test, respectively corrected by means of Tukey’s or Dunn’s multiple comparison test (*P < 0.05; ***P < 0.001).

Preliminary investigation of motor nerve development at 24 hpf showed that synaptic vesicle clusters travel along the motor axons (Fig. S5), and so we used SV2A fluorescence to track these structures in confocal fluorescence maximum projections (Fig. 3A) and measured motor axon length, unbranched axonal length (the distance between the site of axon emergence from the spinal cord and the first branching point), and the number of axonal branches. In comparison with Ctrl, motor axon length and unbranched axonal length were reduced in both transgenic groups, but only the mSod1 embryos showed an increase in the number of axonal branches (Fig. 3B).

**Figure 4:**
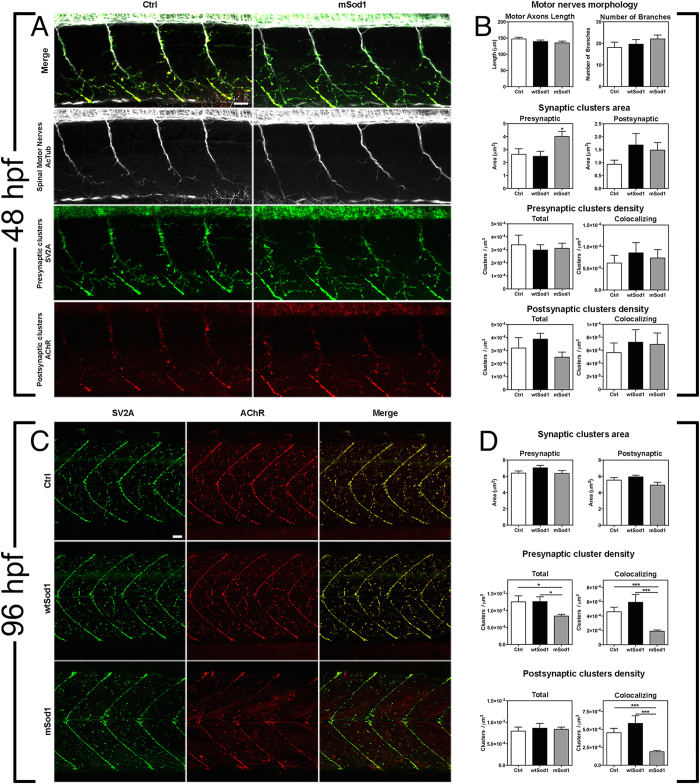
mSod1 embryos and larvae show defects in the clustering of synaptic vesicles and the maturation of neuromuscular junctions. **(A)** Confocal analysis of the 9–13^th^ somite trunk region at 48 hpf. Scale bar: 25 μm. **(B)** There were no differences among the control (Ctrl), wtSod1 and mSod1 embryos and larvae in terms of motor axon length (respectively 147.2 ± 5.2 μm, 139.3 ± 4.6 μm, and 135.1 ± 5.5 μm), the number of branches (18.1 ± 2.5, 19.7 ± 2.1, and 22.1 ± 1.8), or unbranched axon length (28.9 ± 4.8 μm, 6.6 ± 2.1 μm, and 7.9 ± 2.2 μm) (data not shown). The measurements were made in at least four motor nerves of a minimum of 10 embryos for each genotype. Three-dimensional (3D) object-based co-localization analysis (numerical data given in Table 1) did not reveal any differences in the densities of total pre-synaptic or pre-synaptic co-localizing clusters, or in the densities of total post-synaptic or post-synaptic co-localizing clusters, but there was a significant increase in the area of pre-synaptic (but not post-synaptic) clusters in the mSod1 embryos. **(C)** Confocal analysis of the 10–15^th^ somite trunk region at 96 hpf. Scale bar: 25 μm. **(D)** Although the mean area of the pre- and post-synaptic clusters in the different genotypes remained unchanged, 3D object-based co-localisation analysis showed a significant reduction in the density of presynaptic (but not post-synaptic) SV2A clusters in mSod1 NMJs, and a significant reduction in co-localizing pre- and post-synaptic clusters in mSod1 larvae (numerical data given in Table 1).

The same measurements were made in confocal maximum projection images of acetylated tubulin immunofluorescence at 48 hpf (Fig. 4A and Fig. S5), but there were no significant between-genotype differences in motor axon length, the number of branches (Fig. 4B), or unbranched axonal length (not shown). At this stage of development, synaptic vesicles accumulate at the tips of motor nerve branches, whereas AChRs cluster into discernible puncta on the plasma membrane of muscle precursors, and so we used 3D object-based colocalization analysis to evaluate the density of total and co-localizing pre- and post-synaptic clusters in the muscles of the corresponding portion of the trunk. There were no differences in the density of total or co-localizing pre-synaptic or post-synaptic clusters, but there was a significant increase in the area of pre-synaptic clusters in the mSod1 embryos, which showed significantly larger SV2A-positive puncta without any difference in the size of AChR clusters (Fig. 4B and Table 1).

The analyses of pre- and post-synaptic clusters at 96 hpf (Fig. 4C) showed a significant reduction in the density of pre-synaptic clusters in mSod1 larvae (Fig. 4D and Table 1). Moreover, although the density of AChR clusters was similar in the three genotypes, there was a substantial decrease in co-localising pre- and post-synaptic clusters in mSod1 larvae, similar to that observed in adult mutants (Fig. 4D and Table 1), thus suggesting impaired NMJ formation. However, there were no major differences in synaptic vesicle pool or the size of the AChR clusters, as confirmed by ultrastructural analyses of the NMJs (Fig. S6). The small pre-synaptic boutons in the NMJs of zebrafish larvae contained tens of vesicles randomly distributed in the pre-synaptic terminal and mitochondrial profiles, with individual boutons being separated from muscle fibres by a well-defined synaptic cleft. Furthermore, morphometric analyses did not reveal any difference in the area of the NMJ profiles, the overall size of the synaptic vesicle pool, or the size and shape of individual synaptic vesicles. Similarly, the number and characteristics of the pre-synaptic mitochondrial profiles were unchanged (Fig. S6A).

**Figure 5:**
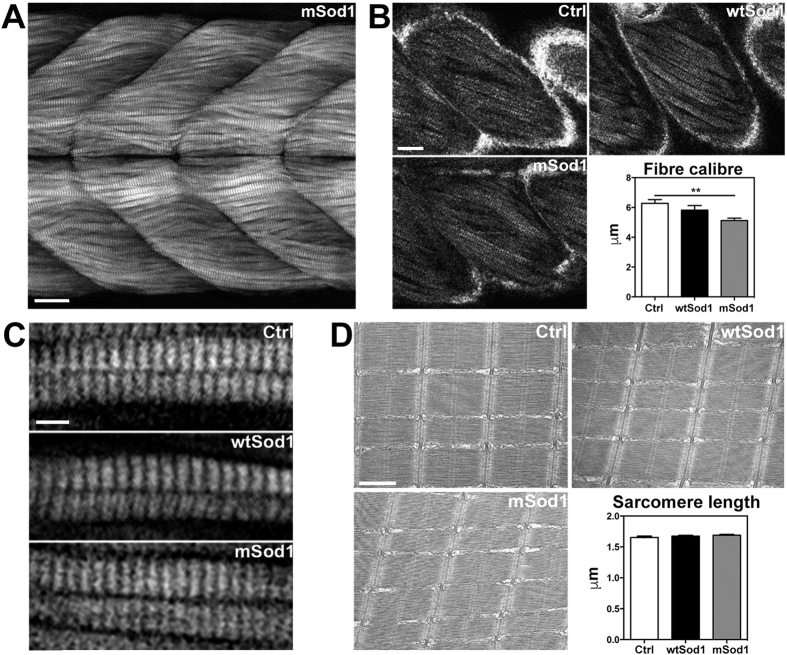
mSod1 larvae show a significant reduction in the calibre of muscle fibres with a preserved sarcomere ultrastructure. **(A)** Maximum projection of myosin second harmonic generation (SHG) signal images in the 12–15^th^ somite region of the trunk of mSod1 larvae. Scale bar: 25 μm. **(B)** Myosin SHG signal images of Ctrl, wtSod1 and mSod1 muscle fibres showing the endogenous myosin signal to quantify muscle fibre calibre (scale bar: 20 μm), which was significantly reduced in the mSod1 than in the control (Ctrl) or wtSod1 larvae (5.13 ± 0.16 μm *vs* 6.28 ± 0.26 μm and 5.82 ± 0.30 μm). The columns indicate the mean value ± SEM in 17 Ctrl, 15 wtSod1 and 18 mSod1 larvae. The data were statistically analyzed using one-way ANOVA and Tukey’s multiple comparison tests (**P < 0.01). **(C)** Detail of myosin SHG signal showing sarcomere organization in Ctrl, wtSod1 and mSod1 muscle fibres. Scale bar: 5 μm. No difference in the length of the sarcomeres in the Ctrl, wtSod1 and mSod1 fibres has been detected. **(D)** Electron micrographs showing the preserved sarcomere ultrastructure in Ctrl, wtSod1 and mSod1 larvae at 96 hpf. Lack of variation in sarcomeres length was confirmed by measuring 100 sarcomeres in two fish per genotype (respectively 1.89 ± 0.03 μm, 1.85 ± 0.03 μm, and 1.90 ± 0.04 μm). The measures were statistically analyzed using one-way ANOVA, corrected by means of Tukey’s post-test.

Muscle precursors complete their differentiation at 96 hpf, and appear as individual fibres with a well-organized contractile apparatus. We analyzed muscle fibre calibre and sarcomere organization in a significant portion of lateral muscle using second harmonic generation (SHG) signal microscopy, and ultrastructurally characterized the sarcomeres and muscle mitochondria using transmission electron microscopy (EM). After acquiring Z-stacks of the myosin SHG signals in the trunk of the larvae (Fig. 5A), we measured fibre calibre in single-plane images of the signals acquired at different depths inside the myotomes, and the length of sarcomeres in a number of different fibres. There was a significant reduction in muscle fibre calibre in mSod1 larvae (Fig. 5B), but no alterations in sarcomere length, and the fine sarcomere structure was equally unaffected (Fig. 5C,D). Interestingly, the EM quantitative analysis of muscle mitochondria showed a significant reduction in the area of mitochondrial profiles (Fig. S6B).

### Sod1 G93R expression leads to precocious motor abnormalities

As the expression of mSod1 is associated with precocious alterations in locomotor network architecture and morphology, we used behavioral tests to assess whether such abnormalities were associated with changes in motor responses. The progressive refinement of locomotor network circuits during the development of zebrafish is accompanied by the appearance of stereotyped motor responses: i) a transient period of alternating spontaneous tail coiling between 17 and 24 hpf; ii) an active response to touch starting from 24 hpf; and iii) the establishment of organized swimming by 96 hpf^13^.

**Figure 6:**
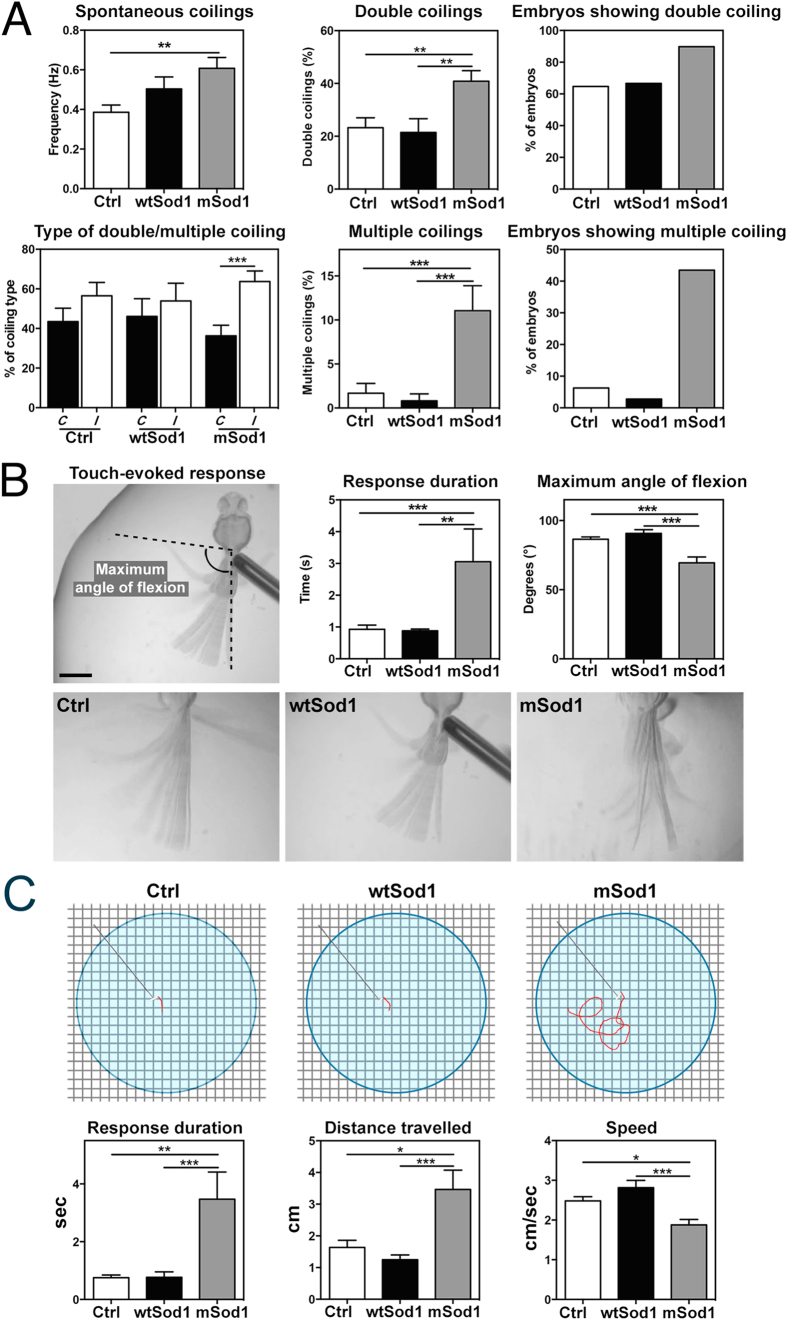
mSod1 embryos and larvae show increased locomotor activity. **(A)** In comparison with controls (Ctrl), mSod1 embryos showed a significant increase in the frequency of spontaneous tail coiling behavior at 20 hpf. The mSod1 embryos showed a significantly higher percentage of both double and multiple coiling in comparison with the Ctrl and wtSod1 embryos. The Ctrl and wtSod1 embryos showed the same percentage of contralateral *(C)* and ipsilateral *(I)* bends of the entire body, whereas the mSod1 embryos showed a significant higher percentage of *I* coilings (numerical data given in Table 2). (**B)** The maximum angle of tail flexion (scale bar: 500 μm) and the duration of touch-evoked responses at 48 hpf. Touch-evoked tail coiling responses lasted significantly longer in the mSod1 embryos, and the maximum angle of tail flexion was significantly less (numerical data given in Table 3). (**C**) Schematic representation of the experimental set-up used to test touch-evoked swimming responses in larvae at 96 hpf, showing a representative response in red. The mSod1 larvae showed significantly longer-lasting evoked swimming responses and travelled significantly further; as these responses consisted of repeated consecutive burst swimming events, the average speed was significantly lower (numerical data given in Table 3).

The frequency of spontaneous tail coiling, the percentage of double and multiple (more than two) bends of the trunk before resting, and the relative percentage of double/multiple coilings consisting of contralateral (*C*, left-right) or ipsilateral (*I*, same side) tail bends were evaluated at 20 hpf (Fig. 6A, Table 2, and Supplementary movies S1 and S2), when the frequency of spontaneous coiling was significantly higher in mSod1 embryos than in Ctrl, but not in wtSod1 embryos. However, the number and rate of double coilings was greater in mSod1 embryos than in either of the other genotypes, and the differences were even more marked in the case of multiple tail coilings. Interestingly, Ctrl and mSod1 embryos executed *C* and *I* bends with the same frequency, whereas the mSod1 embryos executed more double and multiple *I* than *C* bends.

Starting from 24 hpf, when the developing sensory and motor systems integrate, embryos respond to touch with fast, over-the-head coiling of the trunk. An increase in the duration of the touch-evoked responses and a reduction in the maximum angle of tail flexion were observed in 48 hpf mSod1 embryos (Fig. 6B, Table 3, and Supplementary movies S3,S4–S5), and there was an increase in the duration of touch-evoked swimming responses and the distance travelled after every stimulus in 96 hpf mSod1 larvae, as well as a reduction in swimming speed (Fig. 6C, Table 3 and Supplementary Fig. S7).

### Riluzole treatment (5 μM) reverts the motor phenotype in 20 hpf mSod1 embryos and normalizes motor axon length by 24 hpf

**Figure 7:**
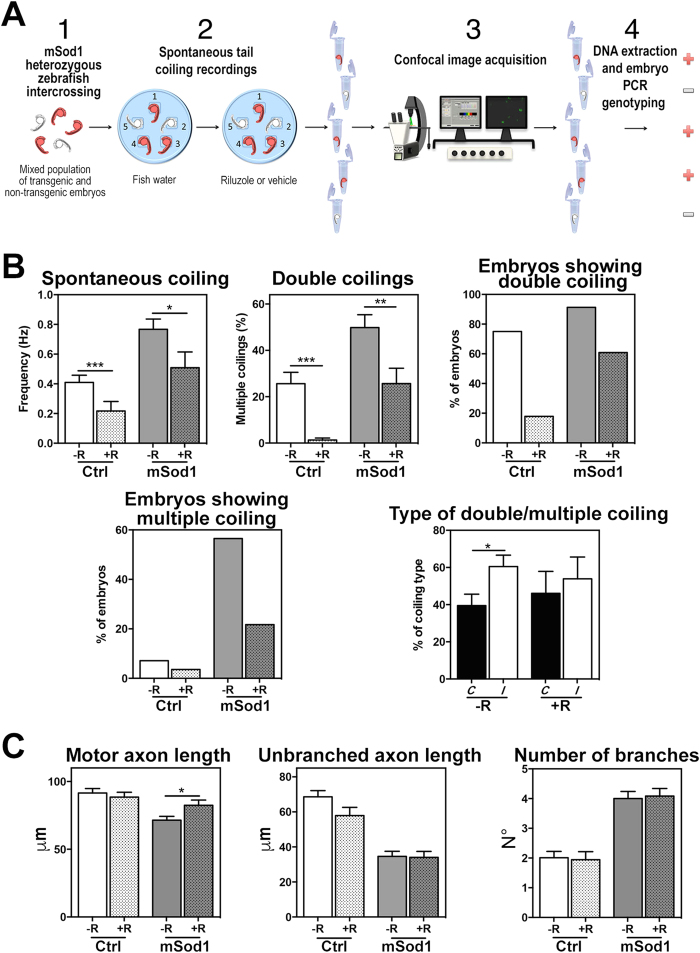
Riluzole treatment reverts the motor phenotype and normalizes motor axon length in mSod1 embryos. **(A)** Protocol used to correlate embryonic behavior (20 hpf) and spinal nerve morphology (24 hpf). Transgenic and non-transgenic embryos from heterozygous mSod1 adults (1) were placed in a petri dish, and spontaneous tail coiling was recorded with (+R) and without riluzole (−R) (2). The embryos were then stained for immunofluorescence, visualized by means of confocal microscopy (3), and PCR-genotyped in order to distinguish the transgenic and control fish (4). **(B)** Riluzole significantly reduced the frequency of spontaneous tail coiling, the percentage of double coiling, and the percentage of double and multiple coiling in both genotypes (Table 4), bringing the frequency of spontaneous coilings, the percentage of multiple tail coilings and the type of multiple tail coilings (the relative percentage of contralateral [*C*] and ipsilateral [*I*] tail bends [39.5% *C* and 60.5% *I* before and 46.13% *C* and 53.87% *I* after riluzole treatment]) to levels comparable with those of the control (Ctrl) embryos before riluzole administration. The columns in each graph indicate the mean value ± SEM of the indicated parameter in 28 Ctrl and 23 mSod1 embryos before and after riluzole treatment. **(C)** Riluzole treatment did not affect motor axon length (91.5 ± 3.3 μm before and 88.5 ± 3.6 μm after), unbranched axon length (68.6 ± 3.6 μm before and 57.9 ± 4.7 μm after) or the number of branches (2.0 ± 0.2 before and 1.9 ± 0.3 after) in the 12–16^th^ somite trunk region of Ctrl embryos, but significantly increased motor axon length (71.44 ± 2.9 μm before and 82.4 ± 3.8 μm after) without affecting unbranched axon length (34.6 ± 2.9 μm before and 34.04 ± 3.4 μm after) or the number of branches (4.0 ± 0.2 before and 4.1 ± 0.3 after) in the same region of mSod1 embryos. The columns in each graph indicate the mean value ± SEM of the indicated parameter in 11 Ctrl and 22 mSod1 embryos treated with riluzole, and respectively 28 and 33 untreated embryos. The measures were statistically analyzed using an unpaired Student *t*-test (see Table 4).

The only four types of spinal neurons active by 20 hpf are synchronized in a spinal network solely by means of electrical coupling, and undergo periodic depolarizations that depend on the persistent I_NaP_ sodium current, which is selectively inhibited by riluzole^16^. We therefore tested whether the aberrant spontaneous coiling phenotype of mSod1 embryos was related to alterations in the activity of this current by studying the effects of 5 μM riluzole on locomotor phenotypes and locomotor network architecture^16^,^19^. To do this, we developed a protocol that allowed us to correlate 20 hpf behavioral responses and 24 hpf spinal nerve morphology in the same embryo (Fig. 7).

After intercrossing heterozygous mSod1 zebrafish to generate a mixed population of transgenic and non-transgenic embryos, we measured spontaneous tail coilings by placing 20 hpf embryos in numbered niches carved in a petri dish and exposing them to riluzole or vehicle solution. Afterwards, each embryo was fixed at 24 hpf and stained for immunofluorescence, and examined through a confocal microscope before being identified by means of PCR on the basis of the presence of the transgene (Fig. 7A). Riluzole treatment significantly reduced the frequency of spontaneous tail coiling in both Ctrl and mSod1 embryos, and reduced the number and rate of double and multiple coilings (Fig. 7B and Table 4); it also balanced the relative percentages of *C* and *I* tail bends in the mSod1 embryos, which otherwise preferentially bend their tails on the same side of the body (Fig. 7B). Riluzole therefore brought global spontaneous coiling frequency, the percentage of double tail coilings and the type of double/multiple tail coilings (the relative percentage of *C* and *I* bends) of mSod1 embryos to levels that are comparable with those recorded in Ctrl embryos before riluzole administration (Fig. 7B).

Finally, confocal fluorescence maximum projection images showing SV2A signals were used to measure axon length, unbranched axonal length and the number of branches. Although riluzole treatment did not affect Ctrl embryos, its administration significantly increased mSod1 motor axon length, but did not change the other parameters (Fig. 7C).

### Spinal neurons in mSod1 embryos show more frequent spontaneous depolarizations

As riluzole-induced I_NaP_ inhibition can revert the motor phenotype of mSod1 embryos and partially recover their altered motor nerve morphology, we looked for potential alterations in I_NaP_-mediated spontaneous depolarizations in the intact spinal network of living embryos using the Mermaid FRET-based voltage biosensor^20^. Fertilized one-cell eggs microinjected with a vector encoding the biosensor under the control of the pan-neuronal promoter HuC^21^ made it possible to obtain sufficient biosensor mosaic expression in a limited population of identifiable 20 hpf neurons (Fig. S8A), and assess their electrical behavior in terms of FRET ratio changes (Fig. S8B).

**Figure 8:**
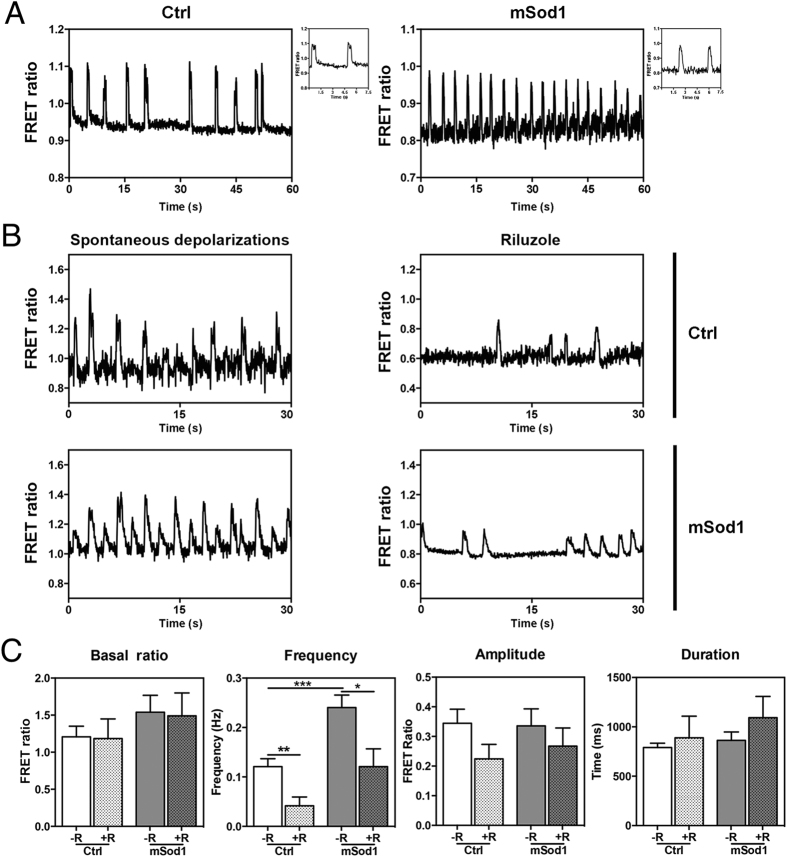
Riluzole treatment reduces spontaneous high-frequency depolarizations in the spinal motor neurons of mSod1 embryos. **(A)** Representative examples of spontaneous mean FRET ratio changes in a control (Ctrl) and mSod1 motor neuron during a one-minute recording (the biosensor FRET ratio increases when membrane potential increases). Insets: detailed morphology of two depolarizing events. **(B)** Representative traces showing the FRET ratio changes recorded in the same Ctrl and mSod1 motor neurons before and after five minutes’ riluzole administration. **(C)** In comparison with Ctrl, the mSod1 motor neurons showed a significant increase in the frequency of spontaneous depolarizations (Ctrl: 0.12 ± 0.02 Hz; mSod1: 0.24 ± 0.03 Hz), but there was no difference in the basal FRET ratio (Ctrl: 1.21 ± 0.14; mSod1: 1.54 ± 0.23), or the amplitude (Ctrl: 0.34 ± 0.05; mSod1: 0.34 ± 0.06) or duration of depolarization (Ctrl: 791 ± 44 ms; mSod1: 863 ± 86 ms). The statistical analyses showed that riluzole did not affect the motor neurons’ basal FRET ratio (Ctrl: 1.18 ± 0.26; mSod1: 1.49 ± 0.31), or the amplitude (0.22 ± 0.05 in Ctrl; mSod1 0.27 ± 0.06) or duration of periodic depolarizations (Ctrl: 889 ± 219 ms; mSod1: 1094 ± 215 ms), but significantly reduced the frequency of the depolarizing events (Ctrl 0.04 ± 0.02 Hz; mSod1 0.12 ± 0.02 Hz). The columns in each graph represent the mean value ± SEM of the indicated parameter in 12 Ctrl and 12 mSod1 motor neurons before and after riluzole administration. The measures were statistically analyzed using an unpaired Student *t*-test (*P < 0.05; **P < 0.01; ***P < 0.001).

Spontaneous depolarizations of spinal motor neurons in mSod1 fish and their non-transgenic siblings were recorded by measuring the basal FRET ratio, the frequency of spontaneous depolarizations, and the duration and amplitude of each depolarization (Fig. 8). The analyses showed a significant increase in the frequency of spontaneous depolarizations in mSod1 embryo motor neurons, whereas no difference in the basal FRET ratio, or in duration or amplitude of spontaneous depolarizations was detected (Fig. 8A,C). Furthermore, riluzole also significantly reduced the frequency of spontaneous depolarizations in the motor neurons of Ctrl embryos (Fig. 8B,C).

**Figure 9:**
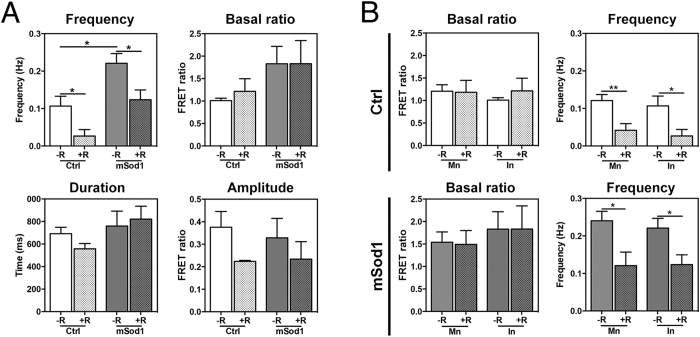
Riluzole treatment reduces spontaneous high-frequency depolarizations in the spinal interneurons of mSod1 embryos. **(A)** The basal FRET ratio, and the frequency amplitude and duration of the depolarizations recorded in type 1 interneurons (T1) in riluzole-treated (+R) and untreated (−R) Ctrl and mSod1 embryos. There were no differences in the basal FRET ratio of T1 in Ctrl and mSod1 embryos before (1.01 ± 0.05 *vs* 1.83 ± 0.39) or after riluzole administration (Ctrl: 1.22 ± 0.28; mSod1: 1.83 ± 0.52). Before riluzole treatment, the T1 in mSod1 embryos showed significantly higher-frequency spontaneous depolarizations than those in Ctrl embryos (0.22 ± 0.03 Hz *vs* 0.11 ± 0.03 Hz), but the frequency was reduced in both after riluzole treatment (mSod1: 0.12 ± 0.03 Hz; Ctrl 0.01 ± 0.01 Hz), although there was no difference in amplitude (Ctrl: 0.37 ± 0.07 [−R] and 0.22 ± 0.01 [+R]; mSod1: 0.33 ± 0.08 [−R] and 0.23 ± 0.07 [+R]) or duration (Ctrl: 692.4 ± 56.8 ms [−R] and 557.7 ± 47.02 ms [+R]; mSod1: 759.8 ± 131.9 ms [−R] and 821.4 ± 114.2 ms [+R]). **(B)** Comparison of the basal FRET ratio and spontaneous depolarization frequency recorded in embryonal spinal cord motor neurons (Mn) and interneurons (In). There was no difference in the basal Ctrl and mSod1 motor neuron and T1 interneuron ratio with (+R) or without riluzole (−R), and no difference between the motor neurons and interneurons of Ctrl and mSod1 embryos in spontaneous depolarization frequency or its reduction after treatment. The basal FRET ratio and frequency (Hz) of spontaneous depolarizations were measured in five Ctrl and 16 mSod1 T1 before and after riluzole administration, and their amplitude (FRET Ratio) and duration (ms) were measured in five Ctrl and five mSod1 T1 with (+R) or without riluzole (−R). The columns in each graph indicate the mean value ± SEM of the indicated parameter. The measures were statistically analyzed using an unpaired Student *t*-test (*P < 0.05; **P < 0.01).

The embryo’s spinal interneurons were classified into three different groups on the basis of their spontaneous depolarization patterns (Fig. S9): type 0, which did not show any spontaneous depolarization before or after riluzole treatment; type 1, which showed spontaneous depolarizations under basal conditions that were negatively regulated by riluzole treatment; and type 2, which did not show any spontaneous depolarizations under basal conditions but developing electrical activity after drug incubation. We concentrated on type 1 interneurons (T1) as they represented the only subtype showing spontaneous activity at such an early developmental stage, and found that they showed significantly more frequent spontaneous depolarizations than Ctrl interneurons but, once again, there was no difference in the basal FRET ratio, amplitude or duration of depolarization. As in the case of motor neurons, riluzole administration significantly reduced the frequency of depolarizations in both mSod1 and Ctrl embryos (Fig. 9A).

Given these similarities, we compared the electrical behavior of interneurons and motor neurons of riluzole-treated and untreated Ctrl and mSod1 embryos. Although it had no effect on the basal FRET ratio, treatment equally affected the frequency of depolarization of both neuron types in both genotypes. In particular, the frequency of spontaneous depolarizations was comparable in both neuron types in both genotypes before and after riluzole administration (Fig. 9B), thus suggesting that changes in the depolarization pattern in mSod1 embryos affected the entire spinal network, and that riluzole reduced spinal neuron depolarization frequency by acting on the entire neuronal network regardless of genotype.

## Discussion

In this study we characterized a mutant Sod1 G93R zebrafish (mSod1) that shares the pathogenic hallmarks of ALS patients and murine models bearing SOD1 mutations. The fish’s ALS-like phenotype was traced back to embryonic/larval stages, which indicated precocious alterations in motor nerve circuitry development that were reverted by 5 μM of riluzole.

Locomotor impairments, spinal cord and muscular atrophy, motor neuron degeneration and NMJs loss, accompanied by alterations in nerve terminals and muscles, are the main traits of the model^17^. Twelwe-month-old fish show the significant alterations in spontaneous locomotor activity previously observed in other SOD1 zebrafish models^22^,^23^.

The onset of clinical symptoms in mutant mice comes with the significant loss of spinal cord somatic motor neurons^24^ and, although the number of spinal motor neurons in mSod1 zebrafish embryos is normal, adult fish are characterized by a reduced number and spinal cord atrophy throughout the trunk. These alterations are accompanied by a significant loss of neuromuscular connections in lateral white muscle and manifest themselves as impaired spontaneous swimming activity, which seems to be due to the degeneration and/or retraction of pre-synaptic terminals as the significant reduction in density is not paralleled by the loss of muscle AChR clusters. Interestingly, as in the case of SOD1G93A mice^25^,^26^, the remaining NMJs of mSod1 zebrafish are apparently no different from those of controls, and so the reason why such a pronounced loss of pre-synaptic terminals is associated with relatively mild locomotor impairment may be the polyinnervation of zebrafish lateral white muscle fibres^11^, which means that probably fewer than 50% of the individual white muscle fibres completely lack nervous input.

A number of studies have shown that glial cells may play a crucial role in the pathogenesis of mammalian ALS, and it is worth noting that we found a significant increase in GFAP in the spinal cords of mSod1 and wtSod1 adult zebrafish, which in line with the gradual increase in astrogliosis that parallels disease progression in mice expressing mSOD1 or wtSOD1^25^.

During the later stages of the disease, the spinal cord of patients and rodent ALS models is characterized by a microglial reaction^9^. We found no difference in the expression of the microglial marker Aif1 in the spinal cord of our transgenic adults, thus supporting previous findings showing that the spinal cords of transgenic mice expressing G93A or wtSOD1 present astrocytosis but not microgliosis in the early stages of disease progression^25^.

Skeletal muscle is one of the tissues affected in ALS patients^26^ (and references therein) and, in line with the loss of motor neuron nerve endings, there was a significant and widespread reduction in the mean calibre of the white fibres of our mSod1 zebrafish, which was particularly severe in the most caudal portion of the body. However, it is interesting that the reduction in fibre diameter was not associated with ultrastructural defects in the contractile apparatus.

The motor axon degeneration and extensive macrophage infiltration of nerve ventral roots, sciatic nerves, and muscles of mice carrying SOD1 mutations^27^ (and our unpublished results) induced us to look for the the presence of activated macrophages and neutrophils around the white lateral muscle fibres of adult mSod1 zebrafish. These were enriched in activated macrophages and neutrophils, which led us to hypothesize that the motor nerve ending retraction and/or degeneration may also determine peripheral macrophage activation in mSod1 zebrafish.

Short and abnormally branching spinal motor neuron axons have been found in zebrafish embryos transiently expressing mutated SOD1^22^,^28^, and it has been shown that the knockdown of C9orf72 and transient over-expression of mutant TARDBP or FUS cause aberrant branching and a significant reduction in the total and unbranched axonal length of embryonic motor nerves^10^,^29^,^30^,^31^. We observed a similar phenotype in 24 hpf wtSod1 and mSod1 embryos, but only the latter showed a significant increase in the number of branches. Although the reduction in motor axon length was comparable with that found in previous analyses of mSod1 embryos^28^, the increase in the mean number of branches per motor nerve was less. This discrepancy may be due to differences in the analyzed time points and/or protein expression levels in the two models (stable transgenesis *vs* transient misexpression). The fact that wtSod1 embryos also showed an aberrant phenotype suggests that a surplus of protein may have a toxic effect on motor axon outgrowth, as previously proposed^22^,^28^, and it is worth noting that, like wtSOD1 transgenic mice and drosophilae^6^,^32^, adult wtSod1 zebrafish also show a slight reduction in locomotor performance and white muscle fibre calibre.

Having found that muscle fibre denervation was associated with a reduction in the density of pre-synaptic inputs in mSod1 adults, we analyzed NMJs in mSod1 embryos by focusing on the establishment of synaptic contacts between motor axons and muscle fibres. We did not observe any differences between the embryos of the three genotypes in terms of the density of migrating vesicle clusters along spinal motor axons, the density of post-synaptic AChR clusters, or the density of co-localizing pre- or post-synaptic clusters, but the mSod1 embryos showed a significant increase in the size of migrating vesicle clusters, which may be due to a perturbation in anterograde axonal transport leading to an increase in the persistency of vesicles along the axons and newly formed branches. Interestingly, similar phenotypes showing defects in organelle accumulation at pre-synaptic terminals have been reported in other mSOD1 models^33^.

This precocious phenotype worsened as development proceeded: at 96 hpf, the difference in the size of synaptic vesicle clusters disappeared, but there was a significant reduction in their density, accompanied by a corresponding reduction in the association of pre- and post-synaptic clusters. Similar results have been obtained in FUS-deficient zebrafish larvae and larvae transiently expressing a mutant FUS protein^34^. This wealth of data supports the hypothesis that mSod1 has a very early toxic effect on the establishment of zebrafish neuromuscular contacts. On the other hand, the size of the AChR clusters, whose formation in this developmental phase is independent of any neuronal inputs^33^ (and references therein), was comparable in all of the embryos.

Given the precocious defects in motor axon outgrowth and NMJ maturation, we investigated whether muscle fibres themselves may be an early primary target of the pathogenic effects of mSod1, and found that mSod1 larvae showed a significant reduction in the calibre muscle fibres which, as in adult fish, was accompanied by the preserved fine structure of the contractile apparatus. Nevertheless, there was a reduction in the size of mSod1 mitochondrial profiles, which suggests that mitochondria may be a disease target even in early developmental stages.

To the best of our knowledge, this is the first study systematically investigating the maturation of motor neuron/muscle fibre contacts that suggests that mutant Sod1-mediated toxicity plays a role in the early development of the apparatus.

Recent studies have shown that the expression of ALS-related genes in zebrafish causes developmental motor impairments^10^,^31^,^34^,^35^, and we found that mSod1 alters the typical stereotyped motor behavior repertoire of embryos and larvae. We hypothesized that the motor phenotype at 20 hpf may be initiated by changes in the electrical properties of spinal neurons and, after measuring the membrane potential of spinal motor neurons and interneurons at this stage, found a significant increase in the frequency of spontaneous depolarizations. This is in line with the findings of various other studies showing pre-symptomatic cortical hyperexcitability in FALS patients^36^,^37^,^38^ and wobbler mouse^39^, altered electrical properties in the spinal cord neurons of transgenic SOD1 G93A mice^40^,^41^,^42^,^43^,^44^ and the iPSC generated by ALS patients carrying SOD1, C9orf72 or FUS mutations^45^, and in zebrafish embryos transiently expressing mutant FUS^34^. This hyperexcitability may also be responsible for the increase in the duration of touch responses at 48 and 96 hpf, although the mutants’ angle of tail flexion (48 hpf) and average speed (96 hpf) were significantly lower and suggest an alteration in trunk muscle activity. Despite this wealth of data supporting the role of hyperexcitability in ALS, a recent study reports that human iPSC-derived motor neurons harbouring ALS-linked mutations exhibit an initial hyperexcitability that progresses into hypoexcitability^46^. Even though it is not possible to directly compare iPSCs to *in-vivo* models, such peculiar behavior is worth further analyses, and we strongly believe that the mSod1 zebrafish model represents a powerful system to monitor, in future studies, spinal network excitability during neuronal development/ageing.

The increased frequency of spontaneous depolarizations in mSod1 embryonic motor neurons and interneurons suggests that the entire locomotor network is characterized by a high degree of intrinsic electrical excitability, which may have contributed to the observed impairment in neuromuscular development, as has also been suggested in the case of the inhibitory glycinergic interneurons in Sod1 G93R embryos^47^. It has recently been demonstrated that ipsilateral caudal (IC) interneurons generate intrinsic spontaneous depolarizations depending on I_NaP_, a persistent pacemaker sodium current that is selectively inhibited by riluzole^16^. We therefore investigated whether an alteration in I_NaP_ activity underlies the increased neuronal excitability by studying the effect of riluzole administration on neuronal spontaneous depolarzations, behavioral responses, and motor nerves morphology. Riluzole reduced the frequency of depolarization events in the spinal cord motor neurons and interneurons at 20 hpf both in Ctrl and mSod1 zebrafish, with the frequency in the latter reaching a value that was similar to that observed in untreated Ctrl fish. As previously hypothesized^42^, this strongly suggests that I_NaP_ is directly involved in mSod1 embryo neuronal hyperexcitability. Interestingly, it has been previously found that riluzole reduces I_NaP_ and rapidly decreases firing frequency in control and mutant Sod1-expressing neurons^40^,^42^. Although I_NaP_ accounts for only 0.8–1% of total neuronal current, it can profoundly affect the behavior of neurons and the neuronal network by enhancing neuronal excitability to near the firing threshold and also because it is essential for spike generation during sustained inputs^19^. As I_NaP_ can be activated at values close to cell resting potential, even small increases can enhance intrinsic cell excitability, alter spike initiation, and amplify the firing rate^19^,^48^.

The role played by I_NaP_ is extremely important in modulating the development of the zebrafish embryo locomotor network under normal conditions because, during the coiling stage (20–25 hpf), the basal membrane potential of IC interneurons is not significantly different from the I_NaP_ threshold, thus suggesting that I_NaP_ pacemaking activity regulates IC depolarization^16^. As IC interneurons directly impinge on a number of motor neurons along the same spinal cord hemi-segment, the increased I_NaP_ activity and IC firing rate in mSod1 neurons led to a higher percentage of multiple ipsilateral tail coilings, a distinctive feature of mSod1 embryos. Furthermore, the fact that riluzole rescued the motor phenotype strongly suggests a direct correlation between neuronal hyperexcitability and this abnormal behavior, and so the increase in the motility of mutant embryos and larvae may be associated with the persistent increase in locomotor network excitability.

Given the numerous parallels between neuronal electrical properties and morphology^41^,^44^,^49^, we also investigated whether the motor axon alterations in mSod1 embryos may be due to their abnormal excitability and firing pattern. Using riluzole to control motor neuron excitability during development, we partially reverted the axonal phenotype as it restored mSod1 motor axon length to control values. This suggests that neuronal excitability plays a role in shaping the neuromuscular apparatus and that riluzole offers a potential pharmacological treatment for aberrant motor nerve morphologies, a result that paves the way to the new field of investigation we are currently pursuing.

In conclusion, our data strongly support the view that zebrafish are a powerful means of studying the mechanisms underlying ALS as they provide a series of ALS-like embryonic phenotypes that can be used to set up unbiased drug screening platforms to identify chemicals that can improve (or exacerbate) them. Our findings also shed light on a new potential mechanism by means of which riluzole may slow disease progression because, together with those of other recent studies^5^, indicate that increased excitability and impaired neuronal activity can be observed very early during development. These early impairments may induce a pathophysiological cascade of events that cannot be reversed by later riluzole treatment, thus explaining the modest increase in patient survival and the lack of effect on disease onset in drug-treated SOD1 G93A mice and suggesting that alternative riluzole treatment protocols should be considered.

## Methods

### Zebrafish (*Danio rerio*) lines

The transgenic *sod1* zebrafish lines used in this study were provided by Dr. Christine E. Beattie of the Centre for Molecular Neurobiology and Department of Neuroscience, Ohio State University, Columbus, USA. The two stable transgenic zebrafish lines respectively express wild-type Sod1 (the os4 line, here referred to as wtSod1) and the G93R mutant of Sod1 (the os10 line, here referred to as mSod1)^17^. In order to be able to identify the construct-positive embryos, the transgenes also carried the zebrafish *heat shock protein 70* (*hsp70*) promoter driving the expression of the red fluorescent protein DsRed. In all of the experiments involving embryos and larvae, we studied the transgenic progeny generated by inter-crossing wtSod1 or mSod1 heterozygous adult zebrafish, and identified the transgenic embryos over-expressing wtSod1 or mSod1 by evaluating DsRed expression at 48 hpf (those younger than 48 hpf were identified by means of PCR genotyping). The non-transgenic progeny were used as controls (Ctrl).

### Fish and embryo maintenance

The fish were maintained at 28 °C on a 14-hour light/10-hour dark cycle; the embryos were collected by means of natural spawning and staged according to Kimmel *et al.*^50^. The embryos used in the whole-mount immunofluorescence experiments were raised in 0.003% 1-phenyl-2 thiourea (Sigma-Aldrich) in order to avoid pigmentation.

Our facility strictly complies with the relevant Italian laws, rules and regulations (Legislative Decree No. 116/92), as confirmed by the authorization issued by the municipality Milan (Art. 10 of Legislative Decree No. 116, dated 27.1.1992). This project was approved by the Italian National Ministry of Health (Animal Welfare Office, Approval No. 162/2015-PR, Legislative Decree No. 26/2014), and all of the procedures were carried out in accordance with the relevant guidelines and regulations.

### Monitoring adult spontaneous locomotor activity

Twelve-month-old wtSod1, mSod1 and Ctrl zebrafish were individually put in a 10.1 × 17.4 cm breeding tank (Tecniplast, Buguggiate, Italy) filled with enough water to guarantee unrestrained swimming of the animal while preventing vertical movements (Fig. S1A). During the 10-minute recording, we measured: the distance travelled by each zebrafish; the time spent resting, evaluated as the percentage of time during which the distance travelled was less than 1 pixel (distance *d* was calculated using the formula: *d* = √(*x*_2_ − *x*_1_)^2^ + (*y*_2_ − *y*_1_)^2^, considering (x_1_, y_1_) the coordinates of the fish at time 1, and (x_2_, y_2_) the coordinates at time 2); and speed, calculated as the ratio between the total distance travelled and the time of effective motion obtained by subtracting the time spent resting from the total time of locomotor activity monitoring (Fig. S1B).

### Externally visible anatomy and body weight evaluation

Twelve-month-old transgenic and Ctrl fish were weighted, photographed and measured, and the typical traits of the zebrafish anatomy were compared: standard length (SL), length between the operculum and caudal peduncle (LOCP), and breadth at the anterior of the anal fin (HAA) (Fig. S2A).

### Histological analyses

Twelve-month-old wtSod, mSod1 and Ctrl zebrafish were anaesthetized using 0.6 mM tricaine (Sigma-Aldrich) in fish water. Their body cavity was opened along the belly, and then fixed in 10% neutral buffered formalin solution (Sigma-Aldrich) for 24 hours. The fish were decalcified in acid-free EDTA 10%, pH 7.2–7.4 (AMRESCO LLC, Solon, OH, USA) for four days, and then transversely sectioned into five segments using their fins as standard anatomical references (Fig. S2B). The first segment (S1) was obtained by making a cut at the level of the operculum; the second segment (S2) went from the operculum to the beginning of the pelvic fin; the third segment (S3) from the pelvic fin to the beginning of the anal fin; the fourth segment (S4) from the beginning to the end of the anal fin; and the fifth segment (S5) from the end of the anal fin to the caudal peduncle. All of the segments were embedded in paraffin in a cranial to caudal orientation, except for the first segment, which was embedded in a caudal to rostral orientation. The samples were processed using an automated tissue processor (Leica Microsystem, Wetzlar, Germany). Each block containing all five segments of a fish was cut into 4 μm sections using a microtome (Leica Biosystems, Nussloch GmbH, Germany), which were stained with hematoxylin and eosin (Mayer’s Hematoxylin, and Eosin G, Diapath, Martinengo, Italy), visualized through an optical microscope, and acquired by means of a digital camera (Leica Microsystem). One or more partially overlapping images covering the entire spinal cord of each fish and four fields containing the white muscle fibres in segments S2–S5 (3–5 sections of each segment) were acquired using a 20x objective, and subsequently fused using Fiji software and the Stitching plugin, an all of the images were analyzed using ImageJ64 software. The area of the spinal cord, the number of the spinal motor neurons, and Feret’s diameter of at least 100 white muscle fibres (the longest distance between any two points along the boundary drawn around transversally cut fibres) were measured in all of the segments of the trunk of each fish (Fig. S2C,D).

### Immunofluorescence of paraffin sections

Formalin-fixed, paraffin-embedded 4 μm sections were deparaffinised in xylene, rehydrated by means of a graded ethanol series, and rinsed in bi-distilled water. Antigens were retrieved using citrate buffer pH 6 1x (Diapath) in a pressure cooker (EMS -Electron Microscopy Sciences, Hatfield, PA, USA). The slides were rinsed, treated with PBS 1x (137 mM NaCl, 2.7 mM KCl, 10 mM Na_2_HPO_4_, 2 mM KH_2_PO_4_) containing 10% normal goat serum (Diapath) for 30 minutes in order to reduce non-specific background staining, and then incubated for one hour at RT with rabbit polyclonal anti-GFAP (glial fibrillary acidic protein, an astrocyte marker) 1:500 (Dako, Glostrup, Denmark) or rabbit polyclonal anti-Aif1 (allograft inflammatory factor 1, a marker of activated macrophages and neutrophils) 1:500 (Wako Pure Chemical Industries Ltd, Osaka, Japan), after which the secondary antibody (AlexaFluor 488 F(ab′)2 Fragment of Goat Anti-Rabbit IgG (H + L), Molecular Probes, Life Technologies Europe BV, Monza, Italy) was added for 30 minutes. Negative controls for each sample were prepared by replacing the primary antibody with PBS 1x containing 10% normal goat serum, and known positive control sections were included in each immunolabelling assay. The immunofluorescence-stained sections were acquired using a TCS SP5 confocal microscope (Leica Microsystems) with a 40x oil-immersion objective, and the images were analysed using ImageJ64 software. The ratio between mean fluorescence intensity in the spinal cord (arbitrary units, a.u.) and the area of the same spinal cord section (μm^2^) was calculated for each GFAP- and Aif1-stained segment. The inflammatory infiltrate in zebrafish lateral muscle was quantitatively measured using ImageJ software in order to calculate the ratio between the surface occupied by the fluorescence signal and the total area under investigation, as well as the ratio between the number of Aif1-positive cells and the total area.

### Immunofluorescence of frozen sections

After sacrifice, the head of each adult fish was removed and the trunk was transversally cut at the level of the pelvic fin, and the two segments obtained were embedded together in Optimal Cutting Temperature (OCT, Tissue-tek, Sakura Finetek Europe B.V. KvK, Leiden, The Netherlands) in a rostral to caudal orientation, and then snap-frozen in liquid nitrogen. The frozen blocks were cryostat cut into transversal sections of 20 μm each (Reichert-Jung 2700-frigocut, Buffalo, NY, USA), and fixed with 2% paraformaldehyde in PBS 1x for 15 minutes at RT, before being permeabilized with PBS-TT (PBS 1x, 0.2% TWEEN20 (Sigma-Aldrich), 0.2% TritonTM X-100 (Sigma-Aldrich), and incubated at RT for 30 minutes in a blocking solution consisting of 3% bovine serum albumin (Sigma-Aldrich) in PBS-TT. The sections were incubated at RT for two hours with the primary rabbit polyclonal antibody anti-SV2A (synaptic vesicle glycoprotein 2A), which stains synaptic vesicles in the pre-synaptic terminal (Sigma-Aldrich, 1:1000 in blocking solution). After rinsing with PBS-TT, the sections were incubated at RT for one hour with the secondary antibody: Alexa Fluor 488 F(ab’)2 Fragment of Goat Anti-Rabbit IgG (H + L) 1:200 and α-Bungarotoxin-Alexa Fluor 555 conjugate 1:500 (Molecular Probes) in blocking solution. After further rinsing with PBS-TT, the stained sections were mounted in ProLong Gold antifade reagent and mounting medium (Molecular Probes), and stored at 4 °C. Negative controls for each sample were prepared by replacing the primary antibody solution with blocking solution.

Z-stacks containing the entire depth of the cryostat section of each sample were acquired using a TCS SP5 confocal microscope with a 63x oil-immersion objective, with 10–15 fields of view of the lateral white muscle of the trunk (containing 7–13 NMJs) being collected for each animal. The z-stacks were analyzed using ImageJ64 software in order to evaluate the percentage of innervation of white fibres in the lateral muscles of the trunk, and the density and size of the pre- and post-synaptic clusters. At least 100 NMJs were analyzed in each animal. In the merged image, we counted the total number of NMJs, the number of post-synaptic clusters facing pre-synaptic clusters in each field and, subsequently, the percentage of innervation and the density of the pre- and post-synaptic specializations.

### Electron microscopy

After sacrifice, the adult fish were rapidly sectioned, and each segment was fixed in 3% glutaraldehyde (EMS), 2% paraformaldehyde in 0.05 M sodium cacodylate (EMS) buffer with 1% sucrose and 1 mM MgSO_4_ (Sigma-Aldrich) for two hours at RT. Subsequently, the lateral muscle of each segment was dissected into small pieces, and further fixed for 48 hours at 4 °C. The samples were rinsed with sodium cacodylate buffer 0.1 M, and post-fixed in 2% osmium tetroxide (EMS) in 0.1 M cacodylate buffer for one hour. After multiple rinses with 0.1 M cacodylate buffer and bi-distilled water, the samples were stained *en bloc* with a saturated solution of uranyl acetate (Sigma-Aldrich) in 20% ethanol for 45 minutes in the dark, and then gradually dehydrated using an ethanol series and propylene-oxide (Sigma-Aldrich), and embedded in an Epon-Spurr resin mixture prepared in accordance with manufacturer’s specifications (TED PELLA Inc., Redding, CA, USA).

Whole zebrafish embryos (24 and 48 hpf) and larvae (96 hpf) were fixed in 2% glutaraldehyde and 2% paraformaldehyde in 0.1 M sodium cacodylate buffer for 12 hours at 4 °C, and then processed following the same protocol. The resin-embedded samples were cut using an ultramicrotome (Leica Microsystem) with a diamond knife (DiATOME, Switzerland), collected on copper grids (EMS), and counter-stained with uranyl acetate solution for 20 minutes, and 1% lead citrate (EMS) for seven minutes, before being observed through a transmission electron microscope (Philips, Eindhoven, The Netherlands). The images acquired using a digital camera (Morada, Olympus, Münster, Germany) were used to make quantitative ultrastructural analyses of adult zebrafish muscle mitochondria in the lateral muscles of the segments S4 and S5. The area, perimeter and circularity of about 100 mitochondria in each animal were measured on electron micrographs acquired at 10500x and 13500x using ImageJ64 software.

The morphological and morphometric analyses of the NMJs at 96 hpf were made using ImageJ64 software and electron micrographs of zebrafish larvae trunk muscle acquired at 34000x. The area of synaptic boutons, the number, density and distribution of synaptic vesicles, and number and morphological features of the mitochondria were evaluated, and the area, perimeter and circularity of NMJ synaptic vesicles and mitochondria were measured^25^. Electron micrographs acquired at 10500x and 13500x were used for the ultrastructural analysis of zebrafish larvae muscles, in which sarcomere length was measured as the distance between two consecutive H-bands in approximately 100 sarcomeres in each fish, and the same micrographs were used to measure the area, perimeter and circularity of about 100 mitochondria.

### Embryo/larva whole-mount immunofluorescence staining

Zebrafish embryos (24 and 48 hpf) and larvae (96 hpf) were fixed in 4% paraformaldehyde and 1% DMSO (Sigma-Aldrich) in PBS 1x, treated with 1 mg/ml collagenase (Sigma-Aldrich) for respectively seven, 15 and 90 minutes at RT, and incubated for at least 12 hours at 4 °C with α-Bungarotoxin-AlexaFluor 555 1:100 in 3% BSA and PBS-TT. After rinsing with PBS 1x, the samples were incubated in acetone (Sigma-Aldrich) at −20 °C for 30 minutes, further washed in PBS-TT, post-fixed with 4% paraformaldehyde in PBS 1x at RT, and then quenched with 50 mM NH_4_Cl (Sigma-Aldrich). After several washes with PBS-TT and three hours’ incubation in blocking solution (3% BSA in PBS-TT), the samples were incubated over night at 4 °C with the primary antibodies, rabbit polyclonal anti-SV2A 1:200 and mouse monoclonal anti-acetylated tubulin (Sigma-Aldrich) 1:500 in blocking solution and, after washing in PBS-TT, incubated over night at 4 °C with the secondary antibodies, AlexaFluor 488 (1:200) and AlexaFluor 633 (1:200) (Molecular Probes) before being washed with PBS 1x and stored at 4 °C. The embryos and larvae were mounted in 1% Low Melting Point Agarose (Sigma-Aldrich) in PBS 1x onto the glass of a 35 mm, glass-bottomed imaging dish (Ibidi, Planegg/Martinsried, Germany), and oriented in order to obtain a lateral view of the trunk. Z-stack images of selected regions of the trunk were acquired using a Leica TCS SP5 confocal microscope and a 20x objective in order to assure the morphological comparison of homogenously developed spinal motor nerves and NMJs. The 24 hpf acquisitions included the region of the trunk ranging from the 12^th^ to the 16^th^ somite above the yolk tube, and then the immediately subsequent region from the 17^th^ to 21^st^ somite; at 48 hpf, we acquired z-stacks of the trunk between the 9^th^ and 13^th^ somite above the yolk tube; and at 96 hpf, the portion of the trunk between the 10^th^ and 15^th^ somite.

### Second harmonic generation (SHG)

The second-harmonic generation (SHG) signal was detected in 96 hpf zebrafish larvae stained and mounted as described above using a Leica TCS SP5 confocal microscope equipped with a multi-photon excitation set-up, and a tuneable, pulsed infrared Ti:Sapphire laser (Chameleon^TM^ Ultra family, Coherent, Milano, Italy) with a wavelength of 900 nm. The signals were collected using an opened pinhole (600 μm) and setting the PMT bandwidth to between 380 nm and 400 nm, and averaged by setting the line average at 3. Z-stacks of each channel were acquired in the order described above using the stack-by-stack mode, a 20x objective, 0.7 numerical aperture (NA) and a 2.5 zoom in order to acquire z-stacks of 310 × 310 μm fields (1024 × 1024 image format, pixel size 303 × 303 nm) with a z-step size of 0.84 μm. The mean fibre calibre of Ctrl, wtSod1 and mSod1 zebrafish larvae was evaluated in 30–60 randomly selected fibres running along the entire stack, and the length of the sarcomeres was calculated as the distance between two minima in the intensity plot profile obtained by tracing a line perpendicularly to the main axis of the fibre (29–60 sarcomeres in several fibres).

### Analysis of the length and axonal branches of spinal motor nerves in 24 and 48 hpf zebrafish embryos

The length, unbranched axonal length (UAL), and number of branches of the motor nerves in segments 9–16 and 17–21 were measured in 24 hpf zebrafish embryos using maximum projections of the SV2A channel and ImageJ64 software. As synaptic vesicles are clustered at motor axon terminals in 48 hpf zebrafish embryos, the same parameters were measured using merged images of the maximum projections obtained from both the SV2A and AcTub z-stacks. The AcTub signal was used to trace the path followed by the motor axons, and SV2A in order to be able to visualize the nerve endings clearly and identify axonal branches.

### Three-dimensional co-localization, and synaptic vesicle and AChR cluster size analyses

The degree of development of NMJs in 48 hpf Ctrl, wtSod1 and mSod1 zebrafish embryos and 96 hpf larvae was evaluated using three-dimensional (3D) co-localization analysis, and synaptic vesicle and muscle AChR cluster density (including the percentage of co-localizing SV2A and AchR clusters) were measured using object-based co-localization analysis with the ImageJ64 Plugin JACoP. The SV2A and AChR stacks were cropped in order to isolate a muscle portion of the acquired volume (aided by SHG myosin signal in the case of the 96 hpf larvae) and, after removing the fluorescence signal due to the developing sensory system, a mean 0.5 filter was used to remove noise. The object-based co-localization analysis was made by working on centre-particle coincidence, in which cluster centres are identified as centres of mass, and the mean size of SV2A and AChRs clusters was calculated.

### Spontaneous tail coiling analysis

Spontaneous tail coiling behavior was evaluated in 20 hpf embryos, after testing each for the lack of a touch-evoked coiling response by means of gentle sensory stimulation. Five embryos at a time were dechorionated and transferred to fish water in a 3.5 mm round petri dish with five niches. Tail coiling was detected at RT during a 5-minute videorecording (time resolution 45 frames/sec) obtained using a digital camera mounted on a stereomicroscope (Leica Microsystems) and for each embryo, a record was made of the frequency of spontaneous tail coiling, the percentage of multiple or complex coilings (i.e. coilings consisting of two or more repeated bends of the trunk before returning to the resting position) and the relative percentages of multiple/complex coilings consisting of contralateral left-right bends of the entire body or ispilateral bends. The effect of riluzole (Sigma-Aldrich) or vehicle (DMSO) on spontaneous tail coilings was tested by gently removing the fish water in order to keep the embryos in the same position, and then adding fish water containing 5 μM riluzole or 0.2% DMSO for five minutes before recording another 5-minute video and making the same analyses.

### Touch-evoked coiling response analysis

Forty-eight hpf Ctrl, wtSod1 or mSod1 zebrafish embryos were transferred to a 10 mm round petri dish in 1% low-melting-point agarose in fish water at 37 °C and oriented with the dorsal side up and belly facing the bottom of the petri dish. When the agarose solidified, all of the agarose behind the yolk ball was gently removed, and a drop of water was added on the top of the fish. At least five tail coilings were evoked by touching the trunk of each embryo above the yolk ball with the tip of a microloader. The fast coilings of the trunk over the head evoked by touch in an embryo were videorecorded at RT (time resolution 45 frames/sec) using a digital camera mounted on a stereomicroscope (Leica Microsystems), and the duration of response and angle of maximum amplitude of tail flexion in each embryo was measured using ImageJ software.

### Touch-evoked burst swimming analysis

Ninety-six hpf zebrafish larvae were transferred to a 10 mm petri dish placed on a 0.5 × 0.5 cm mesh at RT, and their burst swimming responses (fast forward swim with large bend angles) were evoked by touching the trunk behind the head with the tip of a microloader and videorecorded using the high-resolution digital camera of iPhone5 (Apple Inc., Cupertino, CA, US). The duration of response and the distance swum was measured in at least five responses for each embryo.

### Correlations between embryo behavior at 20 hpf and spinal nerve morphology at 24 hpf

As the use of DsRed fluorescence to identify wtSod1 and mSod1 embryos is only feasible starting from 48 hpf, we developed an experimental procedure that makes it possible to identify the embryos at 20 hpf and allows aberrant motor phenotype to be correlated with the morphology of the spinal nerves at 24 hpf. Dechorionated 20 hpf embryos were individually placed in a 3.5 mm petri dish with five niches, and each embryo was tested for spontaneous tail coiling in fish water containing 0.2% DMSO (vehicle) and then in fish water containing 5 μM riluzole. Subsequently, 24 hpf, embryos were fixed in 4% paraformaldehyde in PBS 1x over night at 4 °C, and individually processed for immunofluorescence staining as described above. After confocal microscope image acquisition, each embryo was gently collected from agarose and placed in a 1.5 ml Eppendorf tube for salting-out DNA extraction. The transgenic wtSod1 and mSod1 embryos were identified by means of PCR using a pair of primers spanning a 200 bp region of the DsRed (DsRedFF: 5′-GTAATGCAGAAGAAGACTATGGGCTGGGAG-3′; DsRedRR: 5′-A TGTCCAGCTTGGAGTCCACGTAGTAGTAG-3′). The presence of DNA in each sample was confirmed by PCR using primers specifically amplifying 18S ribosomal subunit DNA (18S_sense 5′-ACCTCACTAAACCATCCAATC-3′; 18S_antisense 5′-AGGAATTCCCAGTAAGCGCA-3′). All of the PCRs were performed using the Mastermix GoTaq G2 master (Promega Italia, Milan, Italy), with 0.4 μM of each primer following a two-step protocol: two minutes of denaturation at 95 °C, and then 35 cycles of 15 seconds’ denaturation (92 °C) and 30 seconds of primer annealing and elongation (55 °C). Unless otherwise indicated, all of the reagents were purchased from Sigma-Aldrich.

### Measurement of membrane voltage of zebrafish embryo spinal neurons using the Mermaid FRET-based voltage biosensor

The Mermaid fluorescence resonance energy transfer (FRET)-based voltage biosensor^20^ (kindly provided by Dr. Hideaki Tsutsui, Laboratory for Cell Function Dynamics, Brain Science Institute, RIKEN, 2–1 Hirosawa, Saitama 351–0198, Japan) was used to measure the spontaneous depolarization of spinal neurons in the intact spinal network of living embryos, with the Mermaid ORF being cloned under the zebrafish HuC pan neural promoter in order to establish the biosensor’s neuron-specific expression^21^. Briefly, the ORF was PCR amplified from the pCS4+ Mermaid plasmid using Pfu Ultra HQ DNA polymerase and the T3 Universal primer and the Mermaid Universal *Sma*I primer (5′TATCCCGGGATTCGACGGTTCAGATTTTA), and the specific PCR product was cloned into the pCMV-SC blunt vector (Strataclone, Agilent Technologies, West Cedar Creek, TX, US). One positive clone (pCMV-SC_Mermaid) was *Sma*I linearized for subsequent insertion of the HuC promoter upstream of the Mermaid ORF. The promoter was synthesized by means of PCR using Pfu Ultra HQ DNA polymerase and zebrafish genomic DNA as template and a pair of HuC-specific primers (HuCprom-forw1_SalI: 5′-GTAGTCGACCAGACTTGTCAAAAGGGTCCA and HuCprom-rev1: 5′-TCCATTCTTGACGTACAAAGATG) spanning a 3150 bp region upstream of the ATG, and the band was cloned into the *Sma*I-linearized pCMV-SC_Mermaid plasmid in order to obtain the final pHuC_Mermaid construct used for the subsequent FRET analysis.

### Imaging set-up for Mermaid biosensor visualization in living embryos: simultaneous detection of donor and acceptor signals

The yolk of one-cell fertilized eggs from mSod1 and Ctrl zebrafish was microinjected with 200 pg of pHuC_Mermaid DNA using a Femtojet microinjector (Eppendorf, Parkway, NY, US). The zygotes were incubated at 28 °C in fish water. At 20 hpf, embryos were mounted in 1% low-melting-point agarose in fish water at 37 °C onto the glass of a 35 mm glass-bottomed imaging dish and gently oriented on their sides. When the agarose solidified at RT, the fluorescence signal was detected using the resonant scanner of a confocal microscope. The fluorescent proteins of the FRET biosensor were simultaneously excited by means of a 488 nm laser line, and the emitted fluorescence (donor channel) was detected between 495 nm and 525 nm in order to collect only the mUKG signal, and the fluorescence emitted by the FRET channel (the fluorescence emitted by the acceptor when the donor is excited) was simultaneously detected between 550 nm and 650 nm. The image fields of 512 × 64 pixels were acquired at a scan speed of 8000 Hz. The changes in spinal neuron voltage under basal conditions were recorded in embryos kept in fish water at RT by acquiring a single xy plane every 30 ms for one minute. The effect of riluzole on membrane depolarization was evaluated in the same neuron five minutes after the addition of fish water containing 5 μM riluzole. In all the acquisitions, gain and offset were optimized at the beginning of the experiment and kept constant throughout the session. At the end of the acquisition, the embryos were checked for the presence of the DsRed DNA by means of a PCR as described above; the negative embryos were considered controls.

The FRET analysis was made using the ImageJ macro Biosensor_FRET created by Robert Bagnell, Pathology & Lab Med UNC-CH in 2010 (www.med.unc.edu/microscopy/resources/imagej-plugins-and-macros/biosensor-fret). The average intensity of an appropriate background region was calculated and subtracted from every pixel of every image and used to process both the donor and FRET stack, after which the images were masked, setting the grey-scale threshold in such a way that the background intensity was zero. The plug-in subsequently started photobleaching correction based on the ratio of the change in the mean brightness of the thresholded parts of the cells over time. The mean FRET and donor fluorescence intensity values were normalized to the first value in each set, and the ratio of the normalized values was calculated for every time point using the formula (FRET mean)^tx^/(FRET mean)^t1^/(Donor mean)^tx^/(Donor mean)^t1^. Subsequently, the raw FRET/Donor ratio stack was calculated, each image was multiplied by the inverse of the curve value at its respective time point in order to correct for the amount of photobleaching, and then the final FRET/Donor ratio was calculated and photobleaching correction was applied in order to obtain a curve showing the normalized FRET ratio changes (y-axis) over time (x-axis), and a double exponential curve was fitted to the data. The normalized FRET ratio calculated for each time point and the fitting curve were then multiplied by the effective FRET ratio calculated at t1 as follows: (FRET mean - FRET background)/(Donor mean - Donor background), where FRET and Donor mean intensity is the mean fluorescence intensity calculated in the same region of interest (ROI) drawn around the cell, and the FRET and Donor background is the mean fluorescence intensity calculated in a ROI of the field without the fluorescent sample. The basal membrane ratio of each neuron was calculated as the mean ratio value calculated from the fitting curve, and the frequency, amplitude and duration of depolarization in those presenting periodic depolarizations were calculated using GraphPad Prism^®^ 6.0c software.

### Statistical analyses

The results were statistically analyzed using GraphPad Prism 6.0c software. Comparisons of two groups were made using an unpaired Student *t* test. When more than two groups were compared, one-way analysis of variance (ANOVA) was used and then corrected by means of Tukey’s or Dunn’s post-test depending on whether the distribution of the values was Gaussian or not. Two-way ANOVA and Bonferroni’s post-test were used to study the effect of mSod1 expression on spinal cord area, and motor neuron number and fibre calibre along the zebrafish trunk. Mean values were considered statistically different when P < 0.05.

## Additional Information

**How to cite this article**: Benedetti, L. *et al.* I_NaP_ selective inhibition reverts precocious inter- and motorneurons hyperexcitability in the Sod1-G93R zebrafish ALS model. *Sci. Rep.* **6**, 24515; doi: 10.1038/srep24515 (2016).

## Supplementary Material

Supplementary Information

Supplementary Movie S1

Supplementary Movie S2

Supplementary Movie S3

Supplementary Movie S4

Supplementary Movie S5

## Figures and Tables

**Table 1:** Characterisation of pre- and post-synaptic clusters in 48 and 96 hpf embryos.

		Synaptic cluster area (μm^2^)		Presynaptic cluster density clusters/μm^2^		Postsynaptic cluster density clusters/μm^2^	
		Pre	Post	Total	Colocalizing	Total	Colocalizing
48 hpf	Ctrl (n = 10)	2.64 ± 0.43^(1)^	0.94 ± 0.16	3.39 ± 0.74 × 10^−4^	0.62 ± 0.18 × 10^−4^	3.20 ± 0.80 × 10^−4^	0.56 ± 0.15 × 10^−4^
	wtSod1 (n = 10)	2.48 ± 0.39^(1)^	1.68 ± 0.44	2.99 ± 0.40 × 10^−4^	0.86 ± 0.24 × 10^−4^	3.88 ± 0.49 × 10^−4^	0.72 ± 0.19 × 10^−4^
	mSod1 (n = 13)	4.02 ± 0.39^(1)^	1.48 ± 0.29	3.11 ± 0.40 × 10^−4^	0.74 ± 0.19 × 10^−4^	2.50 ± 0.39 × 10^−4^	0.69 ± 0.18 × 10^−4^
96 hpf	Ctrl (n = 19)	6.46 ± 0.34	5.15 ± 0.22	1.25 ± 0.02 × 10^−3 (2)^	0.46 ± 0.01×10^−3^	0.79 ± 0.01 × 10^−3 (3)^	0.45 ± 0.01 × 10^−3 (3)^
	wtSod1 (n = 12)	7.33 ± 0.39	6.09 ± 0.28	1.26 ± 0.01 × 10^−3 (2)^	0.59 ± 0.11 × 10^−3^	0.86 ± 0.11 × 10^−3 (3)^	0.58 ± 0.01 × 10^−3 (3)^
	mSod1 (n = 16)	6.24 ± 0.43	5.09 ± 0.40	0.84 ± 0.01 × 10^−3 (2)^	0.19 ± 0.01 × 10^−3^	0.84 ± 0.01 × 10^−3 (3)^	0.19 ± 0.01 × 10^−3 (3)^

Mean values ± SEM. The measures were statistically analyzed using one-way ANOVA, and corrected by means of Tukey’s multiple comparison post-test or the Kruskal-Wallis test and Dunn’s multiple comparison procedure. ^(1)^*p = *0.0093.*^(2)^*p = *0.0145.*^(3)^***p < *0.0001. n* = *number of embryos/larvae.*

**Table 2:** Frequency and characterisation of spontaneous tail coiling in zebrafish embryos at 20 hpf.

	Frequency of spontaneous coiling (Hz)	Double coiling (%)	Embryos showing double coiling (%)	Type of double/multiple coiling (%)		Multiple coiling (%)	Embryos showing multiple coiling (%)
				Ipsilateral	Contralateral		
Ctrl (n = 47)	0.39 ± 0.04	23.24 ± 3.75	64.7	56.5	43.5	1.67 ± 1.11	6.3
wtSod1 (n = 36)	0.5 ± 0.06	21.44 ± 5.2	66.7	53.8	46.2	0.8 ± 0.8	2.8
mSod1 (n = 46)	0.61 ± 0.05**^(1)^	40.84 ± 4.01**^(2)^	89.8	63.7***^(4)^	36.3 ***^(4)^	11.04 ± 2.84***^(3)^	43.5

Mean values ± SEM. The measures were statistically analyzed using one-way ANOVA or the Kruskal-Wallis test, and corrected by means of Tukey’s or Dunn’s multiple comparison test. The relative percentage of contralateral vs ipsilateral bends was calculated using Student’s *t*-test. ^(1)^**p = 0.0066 vs Ctrl. ^(2)^**p = *0.0075 vs Ctrl.*^(3)^***p < *0.0001.*^(4)^***p = *0.0005 (ipsilateral vs contralateral).* n = *number of embryos.*

**Table 3:** Touch-evoked motor responses at 48 and 96 hpf.

48 hpf touch-evoked response			96 hpf touch-evoked burst swimming			
Genotype	Duration (sec)	Angle (°)	Genotype	Duration (sec)	Distance (cm)	Speed (cm/sec)
Ctrl (n = 19)	0.93 ± 0.13 ^(1)^	86.49 ± 1.67 ^(3)^	Ctrl (n = 19)	0.75 ± 0.09 ^(4)^	1.64 ± 0.22 ^(5)^	2.48 ± 0.11 ^(6)^
wtSod1 (n = 14)	0.88 ± 0.05 ^(2)^	90.70 ± 2.73 ^(3)^	wtSod1 (n = 19)	0.77 ± 0.19 ^(3)^	1.25 ± 0.15 ^(3)^	2.82 ± 0.19 ^(3)^
mSod1 (n = 16)	3.06 ± 1.03 ^(1)^	69.47 ± 4.17 ^(3)^	mSod1 (n = 19)	3.47 ± 0.94 ^(4)^	3.47 ± 0.61 ^(5)^	1.88 ± 0.14 ^(6)^

Mean values ± SEM. The measures were statistically analyzed using one-way ANOVA or the Kruskal-Wallis test, and corrected by means of Tukey’s or Dunn’s post test. ^(1)^*** p < *0.001.*^(2)^**p = *0.0053.*^(3)^***p < *0.0001.*^(4)^**p = *0.0057.*^(5)^*p = *0.02.*^(6)^*p = *0.0147. n* *= number of embryos/larvae.*

**Table 4:** Frequency of spontaneous tail coiling in embryos at 20 hpf, before and after riluzole administration.

Riluzole	Frequency of spontaneous coiling (Hz)		Double coilings (%)		Embryos showing double coiling (%)		Embryos showing multiple coiling (%)	
	−	+	−	+	−	+	−	+
Ctrl (n = 23)	0.41 ± 0.05	0.22 ± 0.06***^(1)^	25.6 ± 4.88	1.3 ± 0.84***^(3)^	75	17.9	7.1	3.6
mSod1 (n = 28)	0.77 ± 0.07	0.51 ± 0.11**^(2)^	49.8 ± 5.56	25.7 ± 6.61**^(2)^	91.3	60.9	56.5	21.7

Mean values ± SEM. The measures were statistically analyzed using an unpaired Student *t*-test. ^(1)^***p = *0.0005*(−*vs* + *riluzole*). ^(2)^**p < *0.01* (−*vs* +*riluzole*). ^(3)^***p < *0.0001.* n = *number of embryos.*
