# Supplementary material for: INaP selective inhibition reverts precocious inter- and motorneurons hyperexcitability in the Sod1-G93R zebrafish ALS model
Source: Sci Rep. 2016 Apr 15;6:24515. doi: 10.1038/srep24515 (PMC4832213; doi:10.1038/srep24515)
Supplement: Supplementary Information [file srep24515-s1.doc]

SUPPLEMENTARY INFORMATION

**I_NaP_ selective inhibition reverts precocious inter- and motorneurons hyperexcitability in the Sod1-G93R zebrafish ALS model**

Lorena Benedetti^1^, Anna Ghilardi^2^, Elsa Rottoli^1^, Marcella De Maglie^3^, Laura Prosperi^2^, Carla Perego^4^, Mirko Baruscotti^2^, Annalisa Bucchi^2^, Luca Del Giacco^2*♯^, Maura Francolini^1*♯^

^1^Department of Medical Biotechnology and Translational Medicine, University of Milan, Neuroscience Institute, National Research Council (CNR), Via Vanvitelli 32, 20139 Milano, Italy; ^2^Department of BioSciences, University of Milan, Via Celoria 26, 20133 Milano, Italy;

^3^Department of Veterinary Science and Public Health, University of Milan, Via Celoria 10, 20133 Milano, Italy;

^4^Department of Pharmacological and Biomolecular Sciences, University of Milan, Via Trentacoste 2, 20133 Milano, Italy.

^♯^Equal contributors

*Co-corresponding authors:

Luca Del Giacco

**Tel: +390250314807**

**E-mail: luca.delgiacco@unimi.it**

Maura Francolini

Tel: +390250316977

E-mail: **maura.francolini@unimi.it**

**
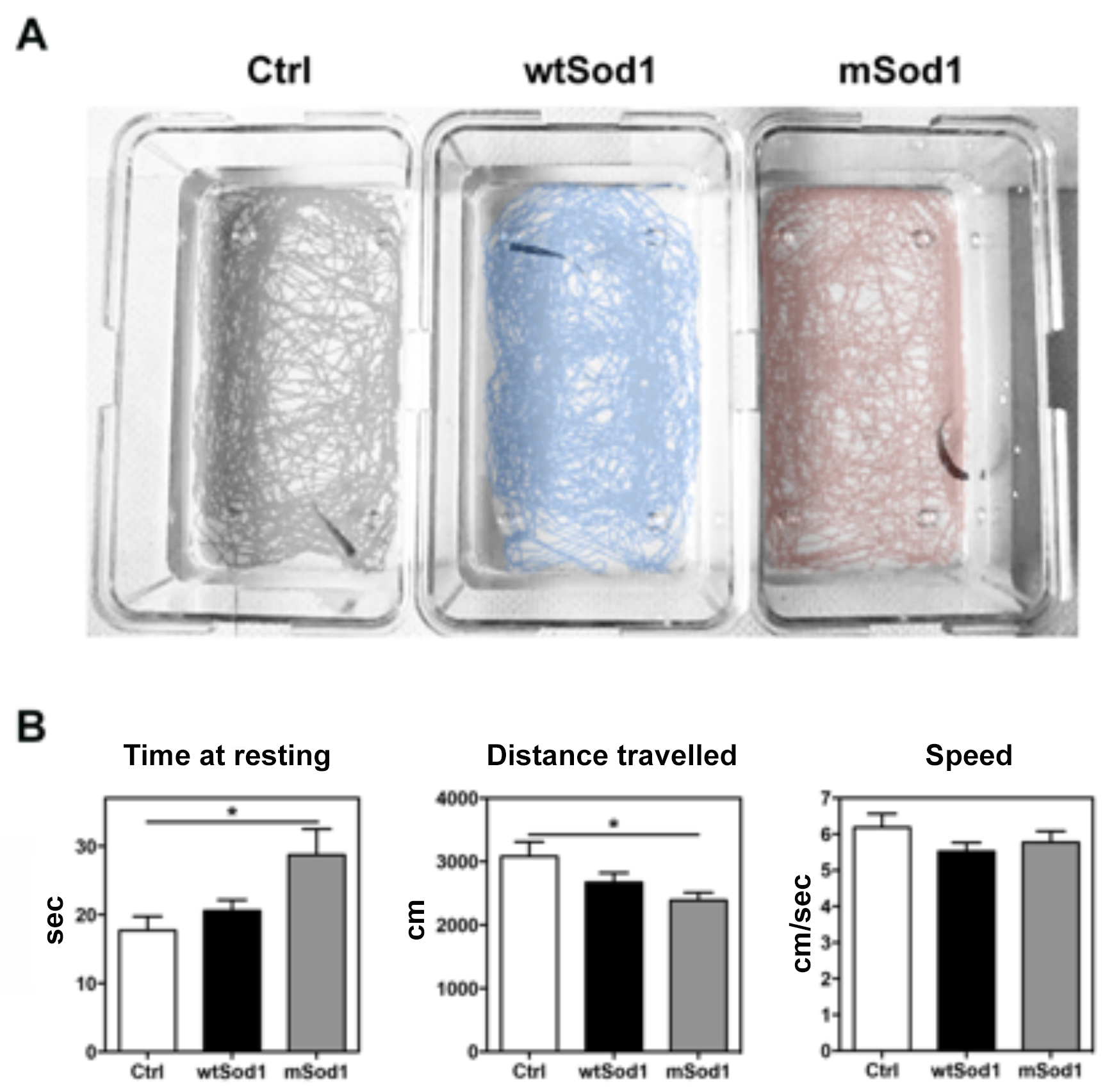
**

**Supplementary Figure S1. Spontaneous swimming activity of 12-month-old zebrafish.** **(A)** Three representative courses swum by Ctrl (grey), wtSod1 (blue), and mSod1 zebrafish (red) of the same sex, obtained using the MTrack2 Fiji Plugin. The movements were simultaneously recorded for 10 minutes in a fish room under fixed and stable lighting, humidity and temperature conditions by means of a digital camera (Olympus D755, Olympus Italia, Milan, Italy). The tanks were positioned close to each other, and the lateral walls were darkened in order to prevent visual cues from affecting the movements of the fish. Each high-resolution film (one frame of 640 x 480 pixels every 0.033 sseconds) was acquired in AVI format using a fixed camera focus and aperture setting, opened using ImageJ 1.48t software (Wayne Rasband, National Institute of Health, USA, http://imagej.nih.gov/ij Java 1.6.0_65, 32-bit), converted into a 8-bit, grey scale image stack, and saved as a TIFF file. The sequence obtained after this process was cropped into three smaller stacks in order to isolate a single breeding tank, and subsequently analyzed to study the movements of one fish at a time using Fiji (ImageJ 1.48k, Wayne Rasband, National Institute of Health, USA, http://imagej.nih.gov/ij Java 1.6.0_65, 64-bit) and MTrack2 Plugin software (Zamani et al., 2011; http://www.haverford.edu/physics-astro/Amador/links/documents/ ImageJHaverflockGuide.pdf). **(B)** The mSod1 zebrafish spent significantly more time resting than the Ctrl (28.73 ± 3.77 *vs* 17.73 ± 2.02 sec; P = 0.014), but not significantly more time than the wtSod1 fish (20.58 ± 1.59 sec), and the same was true in terms of distance covered (2389 ± 122 *vs* 3080 ± 232 cm; P = 0.020 [Ctrl], and 2673 ± 248 cm; P = 0.487 [wtSod1]). However, there was no significant difference in speed: Ctrl: 6.19 ± 0.38 cm/sec; wtSod1: 5.52 ± 0.25 cm/sec; mSod1: 5.77 ± 0.31 cm/sec). Mean values of 15 fish of each genotype in four different experiments, statistically analyzed using one-way ANOVA (*P < 0.05).


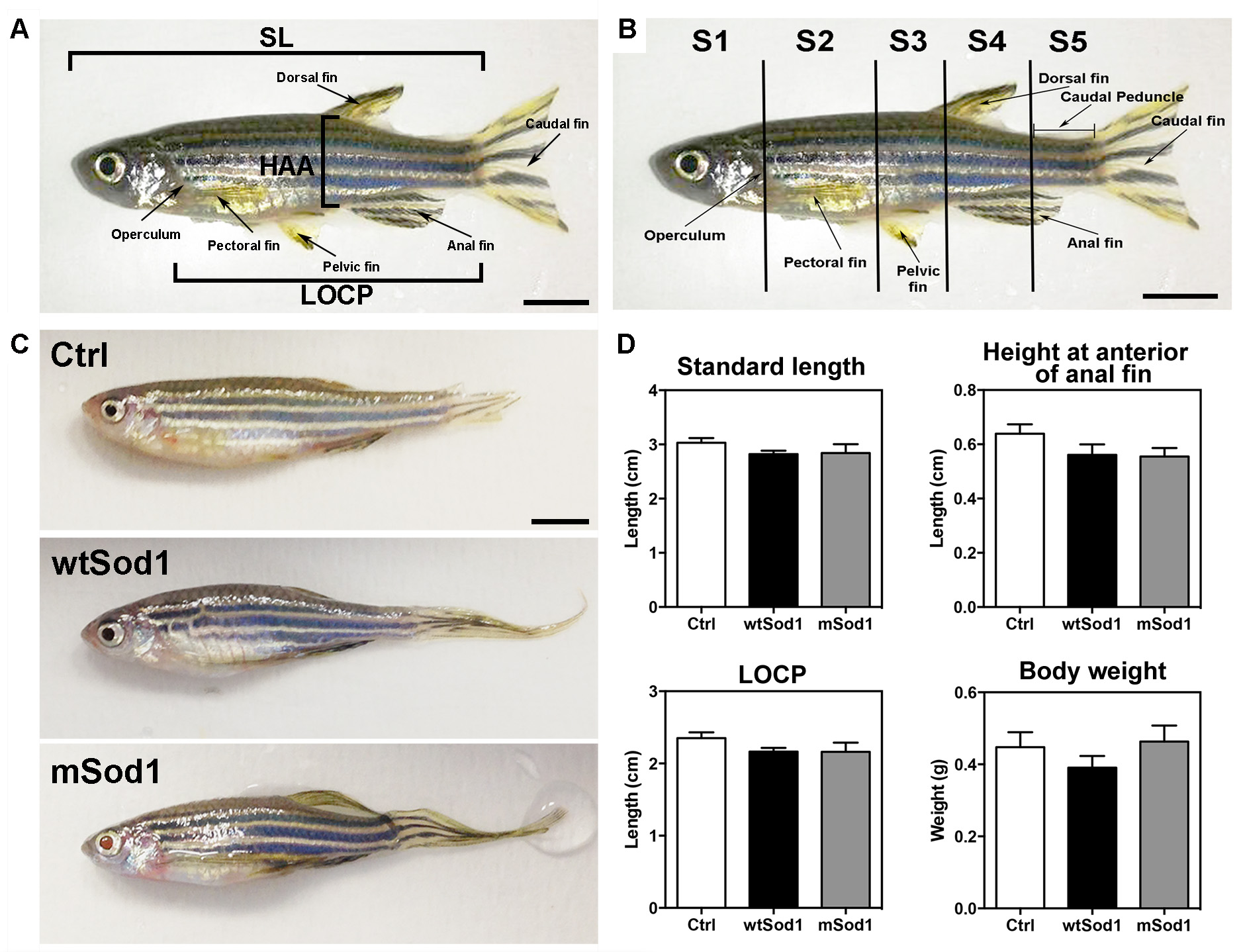


**Supplementary Figure S2. The macroscopic anatomy and body weight of adult zebrafish are not affected by Sod1 over-expression. (A)** Typical traits measured to compare the macroscopic anatomy of the transgenic and Ctrl zebrafish: standard length (SL), the length between the operculum and the caudal peduncle (LOCP), and height at the anterior of the anal fin (HAA). Scale bar: 0.5 cm**. (B)** Each fish was transversely cut into five segments (S1-S5) using the fins as anatomical references. Scale bar: 0.5 cm. **(C)** Representative pictures of 12-month-old zebrafish: an adult of the AB line (Ctrl), and zebrafish expressing wtSod1 or mSod1. Scale bar: 0.5 cm. **(D)** The histograms show SL, HAA, LOCP measured in seven Ctrl, six wtSod1, and seven mSod1 fish, and the body weight recorded in 15 Ctrl, 14 wtSod1, and 14 mSod1 fish. The columns indicate the mean values ± SEM of the indicated parameter, and the results were statistically analyzed using one-way analysis of variance (ANOVA) and the Kruskal-Wallis test, corrected by means of Dunn’s multiple comparison test. There were no significant differences among the three genotypes.
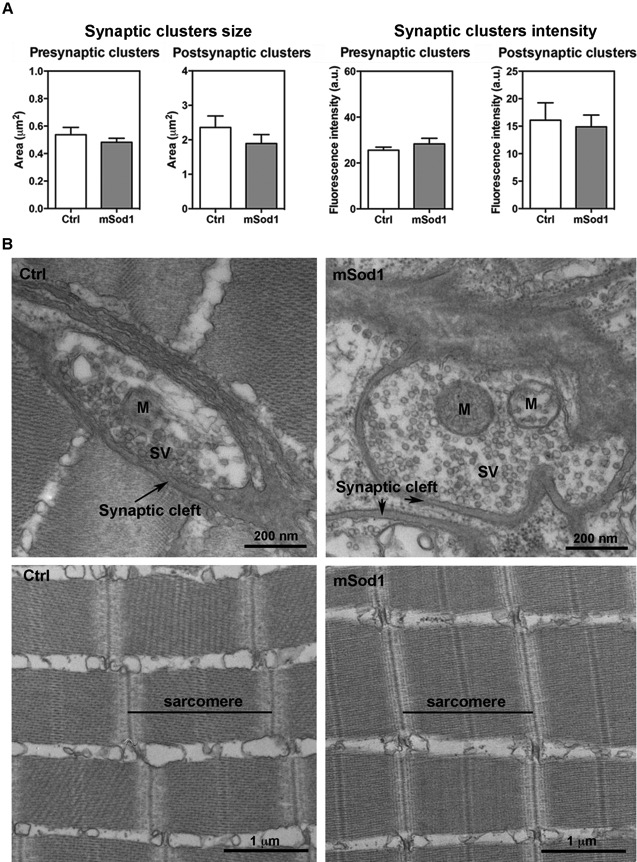


**Supplementary Figure S3. Twelve-month-old zebrafish neuromuscular junction (NMJ) and lateral white muscle structure. (A)** The 3D co-localization analyses of z-stacks covering the entire thickness of the lateral muscle sections of the mSod1 and control (Ctrl) fish did not reveal any significant differences in pre-synaptic (0.48 ± 0.03 μm^2^ *vs* 0.54 ± 0.05 μm^2^) or post-synaptic cluster size (1.89 ± 0.26 μm^2^ *vs* 2.36 ± 0.33 μm^2^), or in the fluorescence intensity of the pre-synaptic (28.34 ± 2.42 a.u. *vs* 25.56 ± 1.39 a.u.) or post-synaptic clusters (14.88 ± 2.16 a.u. *vs* 16.09 ± 3.16 a.u.). The columns indicate the mean values ± SEM of the indicated parameter in five Ctrl and six mSod1 adult zebrafish. The measures were statistically analysed using an unpaired Student *t*-test. **(B)** Representative electron microscopy images of the NMJ (upper panel) and muscle ultrastructure (lower panel) in Ctrl and mSod1 adults. M: mitochondria; SV: synaptic vesicles.


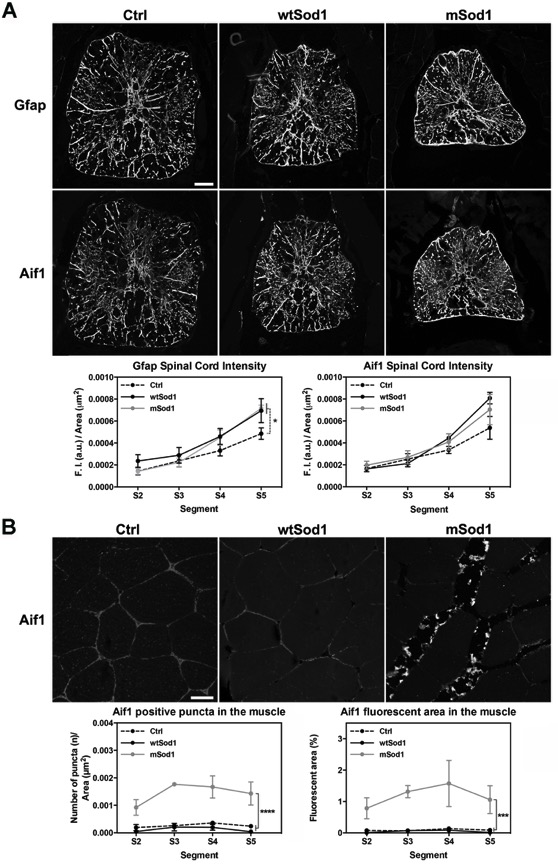


**Supplementary Figure S4. Adult transgenic Sod1 zebrafish show spinal cord reactive astrogliosis but not microgliosis, but only mSod1 fish have activated inflammatory cells in their lateral white muscles. (A)** Reactive astrogliosis and microgliosis were analysed by immunofluorescence in histological sections of 12-month-old zebrafish spinal cord with GFAP (a marker of astrocytes) and Aif1 (a marker of activated microglia in spinal cord and activated macrophages and neutrophils in peripheral tissues), and evaluating the ratio between the mean fluorescence intensity (FI) of both markers and the corresponding spinal cord area (μm^2^) of segments S2-S5. Each point in the graphs represents the mean values ± SEM of the indicated parameter in each segment of seven Ctrl, six wtSod1 and seven mSod1 zebrafish. There was a significant increase in the fluorescence intensity of GFAP in both transgenic spinal cords, but there were no differences in the Aif1 signals. Scale bar: 25 μm. **(B)** Confocal images of Aif1-stained S4 lateral white muscle. Scale bar: 25 μm. The atrophic muscle fibres of mSod1 zebrafish are surrounded by areas enriched in activated macrophages and neutrophils: the graphs show a significant increase in the number of Aif1-positive puncta and the percentage area of lateral white muscle covered by Aif1-positive puncta in all of the examined segments. Each point indicates the mean values ± SEM of the indicated parameter in three adult zebrafish of each genotype. The measures were statistically analysed using two-way ANOVA, corrected by means of Sidak’s post-test. *P<0.05; ***P<0.001; ****P<0.0001.


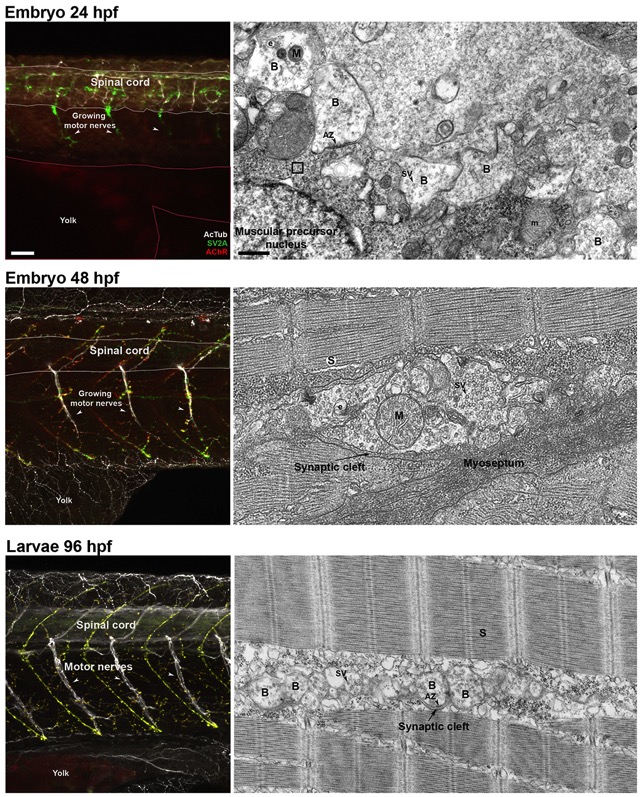


**Supplementary Figure S5. Morphological and ultrastructural changes in developing zebrafish locomotor network.** Control (Ctrl) embryos 24 hpf (upper panels) show short motor axons protruding from the spinal cord entirely filled with synaptic vesicles (stained with anti-SV2A antibodies - green). This outgrowth has a rostral to caudal developmental pattern, and only the more rostral motor nerves show branches. Acetylated tubulin (grey) stains a few spinal interneuronal axonal projections. At this developmental stage, no clusters of acetylcholine receptors (AChRs - red) are visible on muscle fibre precursors. Scale bar: 20 µm. The ultrastructural analysis confirmed that the axonal projections are filled with synaptic vesicles (sv), with immature boutons (B) facing muscle fibres precursors with glycogen-filled cytoplasm (square box) and a poorly organized contractile apparatus. Scale bar: 500 nm. By 48 hpf (middle panels), the motor nerves present a well-organized microtubule network along their entire length, deeply penetrating the trunk along myosepta, and begin innervating the branched muscle fibre precursors. The axons are no longer entirely filled with synaptic vesicles, which are well organized in small clusters at the tips of the axonal branches, and the muscle fibres begin to show visible clusters of AChRs (red). Electron micrographs show pre-synaptic terminals filled with vesicles (sv), mainly located at the periphery of myotomes, and muscle fibres with a well-organized contractile apparatus (S). By 96 hpf (lower panels), the larvae show well-developed, heavily branched motor nerves innervating muscle fibres. Synaptic vesicles are distributed in small clusters at the tips of the axonal terminals, which now face the AChR clusters on muscle fibers (their superimposition generates a yellow signal in the merged image). Ultrastructural analysis reveals small pre-synaptic boutons (B) deeply penetrating into the myotome and innervating well-developed muscle fibres. The confocal images are maximum projections of z-stacks covering half of the trunk. Symbols: AZ: active zone; M: pre-synaptic mitochondria; m: muscle mitochondria; e: endosome.


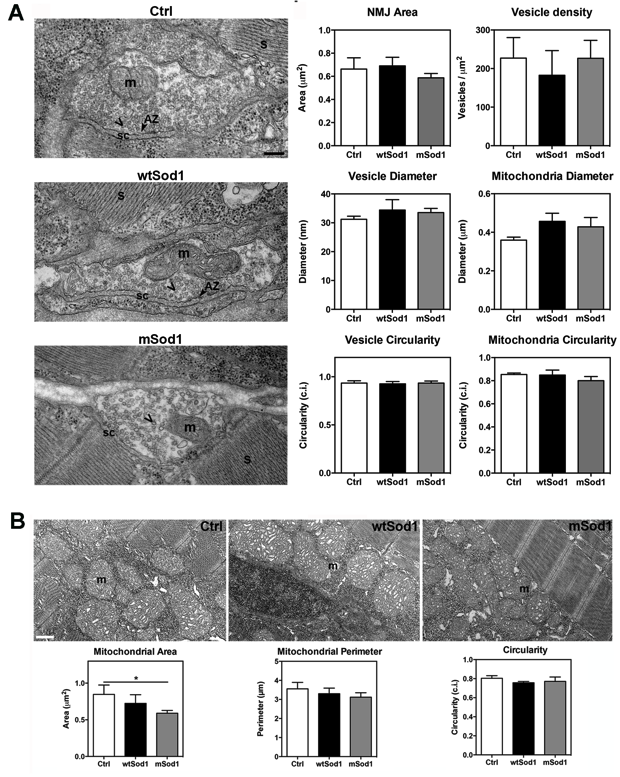


**Supplementary Figure S6. The ultrastructure of mSod1 neuromuscular junctions (NMJs) is preserved at 96 hpf.** **(A)** Electron micrographs showing the neuromuscular junction ultrastructure of 96 hpf control (Ctrl), wtSod1 and mSod1 larvae. The pre-synaptic boutons contained many vesicles (chevron) widely distributed in the pre-synaptic terminals without any particular polarisation, some which are fusing in the active zone (AZ). The pre-synaptic terminals also included mitochondria (m); they are separated from the muscle fibres by a well-defined synaptic cleft (sc). The junctions did not show any clear post-synaptic specialisations. Well-organized sarcomeres (S) were visible in muscle fibres. Scale bar: 200 nm. The morphometric analyses did not reveal any differences in neuromuscular junction area (Ctrl: 0.66 ± 0.10 µm^2^; wtSod1: 0.69 ± 0.07 µm^2^; mSod1: 0.59 ± 0.04 µm^2^), or synaptic vesicle density (Ctrl: 227.10 ± 53.33 n/µm^2^; wtSod1: 182.50 ± 64.10 n/µm^2^; mSod1: 226.70 ± 46.38 n/µm^2^), diameter (Ctrl: 31.20 ± 1.07 nm; wtSod1: 34.42 ± 3.60 nm; mSod1: 33.55 ± 1.44 nm), circularity (Ctrl: 0.94 ± 0.02 circularity index [c.i]; wtSod1: 0.92 ± 0.02 c.i.; mSod1: 0.93 ± 0.02 c.i*.*), area (Ctrl: 588.80 ± 36.45 nm^2^; wtSod1: 695.10 ± 128.90 nm^2^; mSod1: 677.40 ± 52.55 nm^2^; not shown) or perimeter (87.08 ± 3.32 nm in Ctrl, 94.83 ± 10.28 nm in wtSod1, 93.46 ± 4.31 nm in mSod1; not shown) or in mitochondria number (Ctrl: 0.95 ± 0.15; wtSod1: 0.90 ± 0.20; mSod1: 0.80 ± 0.10), diameter (Ctrl: 0.36 ± 0.02 µm; wtSod1: 0.46 ± 0.04 µm; mSod1: 0.43 ± 0.05 µm), circularity (Ctrl: 0.85 ± 0.01 c.i.; wtSod1: 0.85 ± 0.04 c.i.; mSod1: 0.80 ± 0.04 c.i.), area (Ctrl: 0.08 ± 0.01 µm^2^; wtSod1: 0.12 ± 0.03 µm^2^; mSod1: 0.08 ± 0.01 µm^2^; not shown) or perimeter (Ctrl: 1.07 ± 0.06 µm; wtSod1: 1.28 ± 0.14 µm; mSod1: 1.11 ± 0.05 µm; not shown) in the pre-synaptic terminals. The columns represent the mean values ± SEM of the indicated parameter in 30 neuromuscular junctions of three fish of each genotype. The measures were statistically analysed using the Kruskal-Wallis test, corrected by means of Dunn’s multiple comparison procedure. **(B)** Electron microscopy images showing muscle mitochondria (m) morphology in Ctrl, wtSod1 and mSod1 larvae. Scale bar: 500 nm. Muscle mitochondria area was significantly smaller in the mSod1 larvae (Ctrl: 0.89 ± 0.09 μm^2^; wtSod1: 0.82 ± 0.12 μm^2^; mSod1: 0.54 ± 0.04 μm^2^), but there was no significant difference in mitochondria perimeter (Ctrl: 3.66 ± 0.23 μm; wtSod1: 3.44 ± 0.25 μm; mSod1: 2.93 ± 0.19 μm) or circularity (Ctrl: 0.81 ± 0.02 c.i.; wtSod1: 0.79 ± 0.03 c.i.; mSod1: 0.79 ± 0.03 c.i). The measurements refer to one hundred mitochondria in six Ctrl, four wtSod1 and six mSod1 larvae, and were statistically analysed using one-way ANOVA, corrected by means of Tukey’s multiple comparison tests (*P<0.05).

**
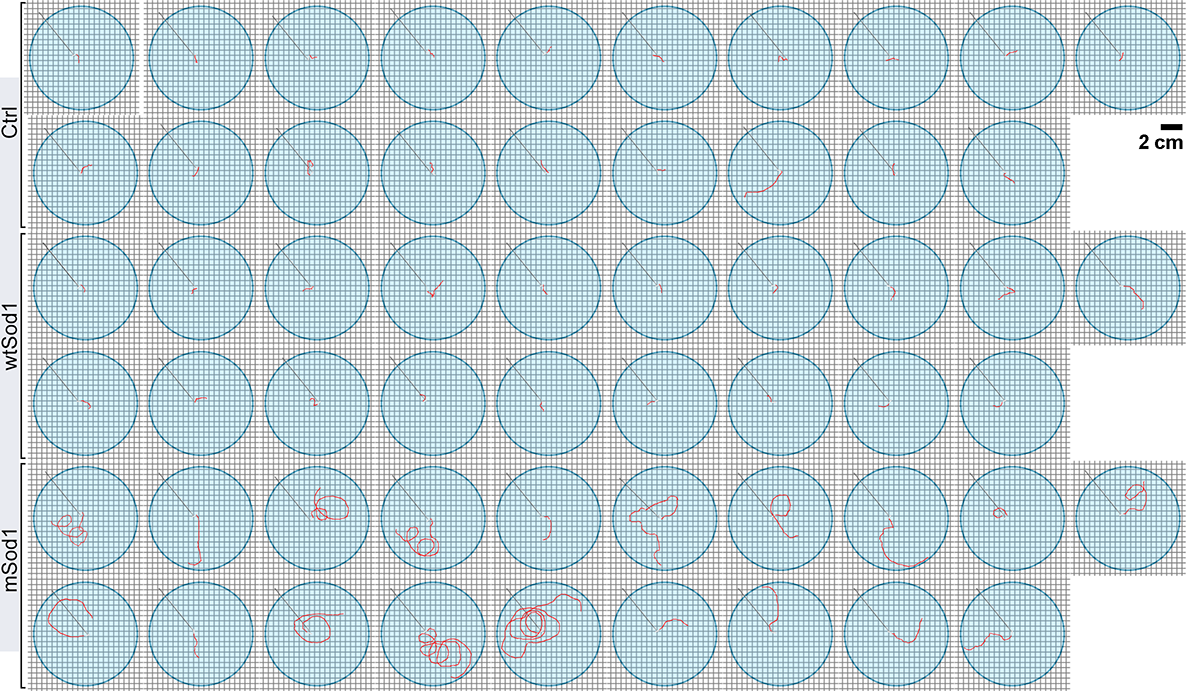
**

**Supplementary Figure S7. Touch-evoked swimming responses in 96 hpf larvae**. Representative traces of control (Ctrl, upper panel), wtSod1 (middle panel) and mSod1 (lower panel) touch-evoked burst swimming in 96 hpf larvae. The mSod1 larvae showed significantly longer-lasting evoked swimming responses and travelled significantly further; as these responses consisted of repeated consecutive burst swimming events, the average speed was significantly lower (see Fig. 6C and Tab. 3).


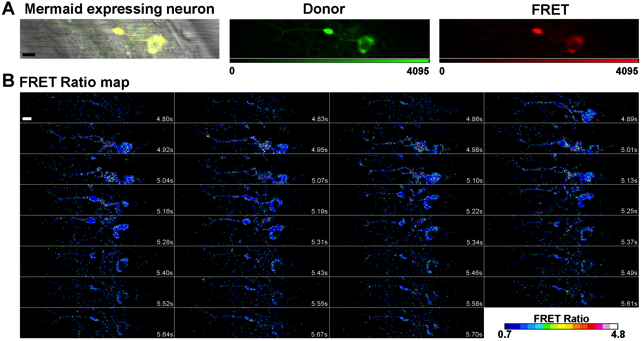


**Supplementary Figure S8. Mosaic expression of HuC:Mermaid vector in a 20 hpf zebrafish spinal motor neuron, and a FRET ratio map of a spontaneous depolarisation event.** **(A)** In order to verify whether the aberrant motor phenotype observed in 20 hpf mSod1 embryos was associated with alterations in the spontaneous depolarisation of spinal neurons, one-cell-stage embryos were micro-injected with the FRET-based voltage biosensor Mermaid ORF under the control of the pan-neuronal promoter HuC. The bright field image merged with the fluorescence signal shows efficient biosensor expression in a spinal motor neuron. The detected donor and FRET channel are also shown. Scale bar: 10 µm. **(B)** The time points on the FRET ratio map of the same motor neuron during a spontaneous depolarisation event shows that the FRET ratio increases with membrane potential. Scale bar: 10 µm.


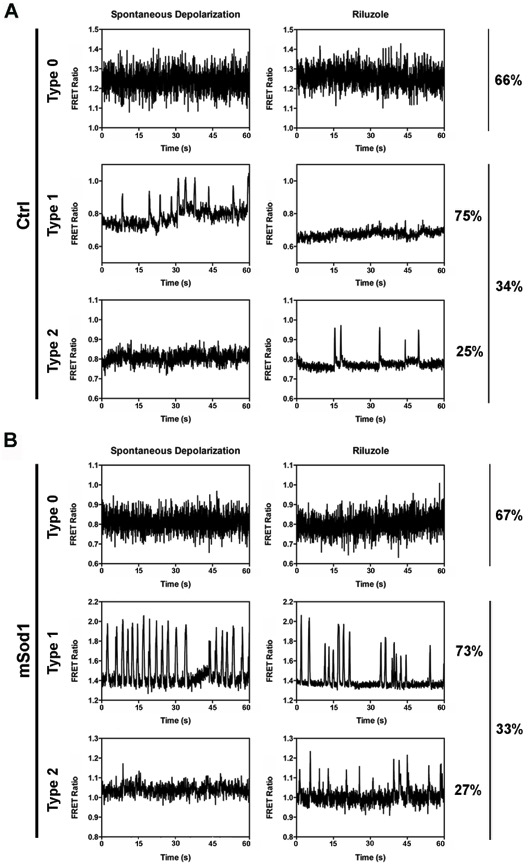


**Supplementary Figure S9. Spinal interneurons in control (Ctrl) and mSod1 zebrafish embryos show different patterns of changes in membrane voltage and respond differently to riluzole treatment.** Representative examples of the FRET ratio changes recorded before and after riluzole administration in type 0, 1, and 2 interneurons. **A)** Most of the cells in Ctrl embryos (66%) do not show periodic depolarisations, and their electrical properties do not change after riluzole treatment (type 0); of the remaining cells, 75% show display periodic depolarisations before riluzole incubation (type 1), and 25% afterwards (type 2). **B)** In mSod1 embryos, 67% are type 0 interneurons, 73% type 1, and 27% type 2.

**Supplementary Movie S1. Spontaneous tail coiling in 20 hpf control embryos**

Representative recording illustrating spontaneous tail coiling in a 20 hpf control embryo.

**Supplementary Movie S2. Spontaneous tail coiling in 20 hpf mSod1 embryos**

Representative recording illustrating spontaneous tail coiling in a 20 hpf mSod1 embryo. Compared to controls, mSod1 embryos showed a significant increase in the frequency of spontaneous tail coiling behavior at 20 hpf and a significantly higher percentage of both double and multiple coiling (see Fig. 6A and Tab. 2).

**Supplementary Movie S3. Touch-evoked coiling response in 48 hpf control embryos**

Representative recording illustrating touch-evoked coiling in 48 hpf control embryos.

**Supplementary Movie S4. Touch-evoked coiling response in 48 hpf wtSod1 embryos**

Representative recording illustrating touch-evoked coiling in 48 hpf wtSod1 embryos.

**Supplementary Movie S5. Touch-evoked coiling response in 48 hpf mSod1 embryos**

Representative recording illustrating touch-evoked coiling in 48 hpf mSod1 embryos. The maximum angle of tail flexion was significantly reduced while individual responses lasted significantly longer in the mSod1 embryos (see Fig. 6B and Tab. 3).
